# Origins of Nitrite and Nitrate Selectivity in Aqueous Electrocatalytic Ammonia Oxidation by a Mononuclear Copper Catalyst

**DOI:** 10.1021/acs.inorgchem.6c00859

**Published:** 2026-05-18

**Authors:** Joel Leitão Nascimento, João Pedro C. S. Neves, Roberto Rivelino, Vitor H. Menezes da Silva, Tiago Vinicius Alves

**Affiliations:** † Departamento de Físico-Química, Instituto de Química, 28111 Universidade Federal da Bahia, Rua Barao de Jeremoabo, Salvador, Bahia 40170-115, Brazil; ‡ Instituto de Física, Universidade Federal da Bahia, Salvador, Bahia 40210-340, Brazil

## Abstract

The electrocatalytic oxidation of ammonia to nitrite and nitrate catalyzed by the mononuclear copper-based complex [Cu­(pyalk)_2_] (pyalk = 2-(pyridin-2-yl)­propan-2-oate) has recently demonstrated remarkable electroselectivity (preferential formation of nitrate or nitrite as a function of the applied potential) and chemoselectivity (water vs ammonia oxidation as a function of pH). However, the mechanistic origin of these selectivities remains unclear. Herein, we provide new insights into the complete electrochemical catalytic cycle of ammonia oxidation (AO) in aqueous media from quantum mechanical calculations. Our results show that the transformation does not proceed through a single oxyl-centered pathway, but through competing oxidation-driven sequences that activate Cu complexes to enable successive N–O bond formation. New mechanistic scenarios are identified in which water participates as a nucleophile, while the ammonia buffer plays a crucial role throughout the catalytic cycle. In addition, ammonia saturation enables participation in the secondary coordination sphere, promoting key intermolecular interactions favorable to N–O bond coupling. Finally, we demonstrate that subsequent electrochemical oxidation steps act as the primary driving forces governing the experimentally observed selectivity. Overall, this work establishes an unprecedented mechanistic framework for copper-mediated ammonia oxidation and provides molecular-level insights into the factors that control selectivity in intricate electrocatalytic nitrogen transformations.

## Graphical abstract

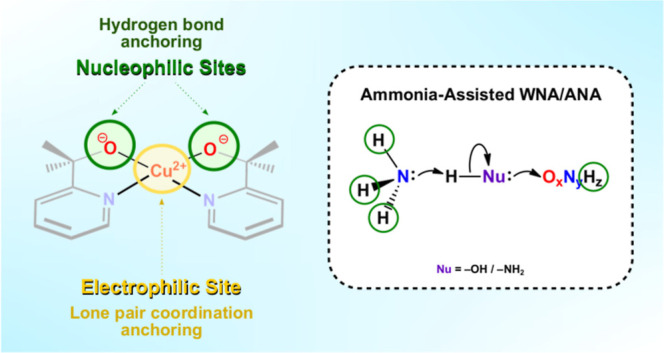

## Introduction

Ammonia is produced on a large scale via the Haber-Bosch process^1^ and has emerged as a promising hydrogen carrier and storage medium, featuring a gravimetric hydrogen content of approximately 17.7%.^2^ It has also gained attention as a carbon-free fuel,^3^ thus providing opportunities for blending it with other fuels to facilitate combustion and enable gradual greener transition strategies.^4,5^ Beyond its role in energy applications, the ammonia oxidation (AO) process, as classically described by the Ostwald process^6^ (as shown in reactions **R1** to **R3**), constitutes the basis for the industrial production of nitric acid (HNO_3_) or nitrate (NO_3_^–^), which are of central importance in fertilizer manufacturing. Despite its extensive industrial deployment over the years, the Ostwald process has notable drawbacks, including operation at high temperatures and pressures, high energy demand, and reliance on noble-metal catalysts.^7,8^ These limitations have inspired new research toward the development of more sustainable approaches, such as electrocatalytic processes using transition-metal complexes, particularly those based on the 3*d* series. Specifically, the selective electrochemical production of NO_2_^–^ and NO_3_^–^ has emerged as an attractive alternative, mainly when operating under milder and catalytic conditions.^9,10^

$$4{NH}_{3}\left(g\right)+5O_{2}\left(g\right)\to 4NO\left(g\right)+6H_{2}O\left(g\right)$$ R1

$$2NO\left(g\right)+O_{2}\left(g\right)\to 2{NO}_{2}\left(g\right)+6H_{2}O\left(g\right)$$ R2

$$3{NO}_{2}\left(g\right)+H_{2}O\left(l\right)\to 2{HNO}_{3}\left(aq\right)+NO\left(g\right)$$ R3

The majority of monometallic molecular catalysts for electrochemical AO, employing complexes with Ru, Fe, Mg, and Cu centers,^11–17^ have been typically directed toward selective three-electron N_2_ formation, often carried out in organic solvents, which minimizes the generation of oxidized nitrogen species (NO_2_^–^ and NO_3_^–^). In this context, Holub et al.^18^ recently demonstrated that certain Ru complexes containing polypyridyl ligands generate small amounts of these oxidized species when catalysis is performed in water, thereby reducing the Faradaic efficiency toward N_2_ production. Complementarily, Roithmeyer et al.^19^ further demonstrated that Ru complexes anchored to electrode surfaces via a heterogenized approach can selectively produce nitrate, highlighting the potential of supported molecular catalysts for controlled ammonia oxidation.

Recently, Brudvig’s group was among the first to report mononuclear copper-based catalysts capable of high selectivity for NO_2_^–^ and NO_3_^–^ formation from electrocatalytic ammonia oxidation.^20,21^ The first homogeneous copper system, reported in 2022,^20^ employed the [Cu­(bipyalk)]^+^ complex (wherein the bipyalk ligand is 2-[(2,2′-bipyridin)-6-yl]­propan-2-ol), as depicted in Scheme 1A. Indeed, the [Cu­(bipyalk)]^+^ complex was shown to be resistant to oxidation under aqueous conditions, enabling highly selective ammonia oxidation over competing water oxidation (WO) in aqueous media. The choice of copper as the metal center was motivated by its abundance in the Earth’s crust and inspired by its biological role in ammonia oxidation.^22,23^ Later on, Brudvig’s group^21^ reported the complex [Cu­(pyalk)_2_] (pyalk = 2-(pyridin-2-yl)­propan-2-oate) as an active catalyst for AO; as shown in Scheme 1B. This complex, previously applied in water oxidation catalysis,^24,25^ demonstrated that pH control via the ammonia buffer can favor chemoselective ammonia oxidation (at pH = 9, as shown in reactions **R4**-**R5**) over water oxidation under aqueous conditions (at pH = 13), thereby expanding the potential of these catalysts in aqueous media. Furthermore, selectivity toward NO_2_^–^ or NO_3_^–^ formation was achieved by varying the applied potential. Specifically, potentials in the range of 1.50–1.60 V favor the thermodynamic product NO_3_^–^, whereas potentials below 1.50 V accelerate NO_2_^–^ formation. However, a detailed mechanistic explanation for these chemo- and electroselectivities, based on the kinetic and thermodynamic features of the key reaction pathways within the electrocatalytic cycle, remains pending.

$${NH}_{3}+2H_{2}O\to {NO}_{2}^{-}+7H^{+}+6e^{-}$$ R4

R5 $${NO}_{2}^{-}+H_{2}O\to {NO}_{3}^{-}+2H^{+}+{2e}^{-}$$

**Scheme 1:**
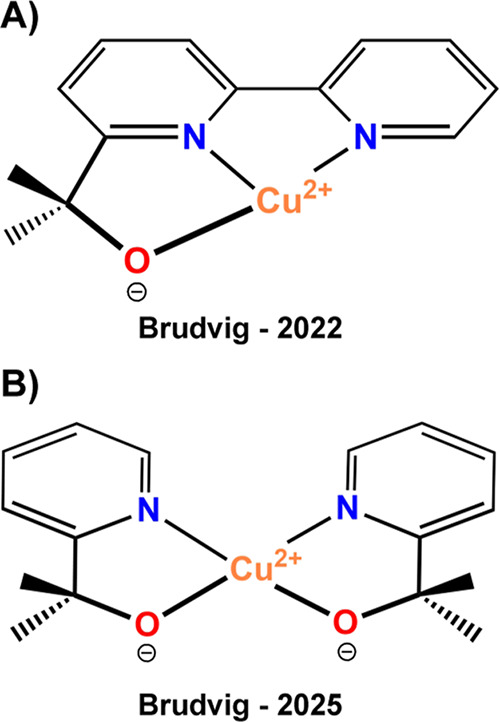
Electrochemical Molecular Ammonia Oxidation (AO) Catalysts Reported by Brudvig’s Group: (A) [Cu­(bipyalk)]^+^ (2022)^20^ and (B) [Cu­(pyalk)_2_] (2025)^21^

In particular, the electrocatalytic cycle of water oxidation catalyzed by [Cu­(pyalk)_2_] has been experimentally and theoretically elucidated, providing crucial mechanistic insights.^24^ For instance, these studies pointed out that water molecules may interact with the anionic oxygen atoms of the ligands through hydrogen bonding. By analogy, similar second-sphere interactions can be proposed to occur during ammonia oxidation involving the [Cu­(pyalk)_2_] catalyst, in which a water molecule coordinated at the equatorial position of a square-pyramidal environment could give rise to an oxyl species, although direct ammonia coordination to the copper center cannot be completely ruled out. This arrangement potentially explains the pH-dependent control of the reaction, directing water nucleophilic attack (WNA) at pH = 13 toward O_2_ evolution, while ammonia nucleophilic attack (ANA) is favored at pH = 9, leading to the formation of NO_2_^–^ and NO_3_^–^.^21^ Furthermore, cyclic voltammetry measurements of the AO process display only a single electrochemical event (1.13 V vs NHE; 1.66 V vs RHE), which has been assigned to the Cu^II^/Cu^III^ single-electron transfer (SET) process, analogously to WO catalysis. Although the authors proposed possible oxidation/activation of the catalyst to enable consecutive N–O bond formation steps, detailed mechanistic information regarding the elementary steps and reaction intermediates responsible for these electrochemical features is absent from the original experimental study.

Indeed, despite thorough experimental investigation previously reported, we believe that a comprehensive computational study of the full electrocatalytic cycle for NO_2_^–^ and NO_3_^–^ production from NH_3_, based on reliable quantum chemical methodologies and encompassing all elementary electrochemical steps, is particularly challenging but essential for understanding of the key features of this electrochemical reaction, especially regarding the selectivity findings from an atomic- and molecular-level perspective. For instance, AO may proceed through multiple reaction manifolds, as outlined in Scheme 2, involving either 7H^+^ and 6e^–^ (Steps A to C/Reaction **R4**) or 9H^+^ and 8e^–^ (Steps A to D/Reactions **R4** and **R5**), in agreement with previous stoichiometric considerations. Scheme 2 provides a global overview of the viable mechanistic pathways for NO_2_^–^ and NO_3_^–^ formation, which are expected to involve multiple proton-coupled electron transfer (PCET) and/or single-electron transfer (SET) events, as well as several copper-containing reaction intermediates featuring different metal oxidation states. Based on other electrocatalytic systems,^24,26,27^ we expect these oxidation steps to constitute the main driving force for each N–O bond-forming event. Preliminary theoretical studies addressing N_2_ formation,^28,29^ combined with our new computational findings, indicate that the first electrochemical event (Step A) involves catalyst activation followed by ammonia nucleophilic attack (ANA) or water nucleophilic attack (WNA) pathways, based on the formal 2H^+^/2e^–^ stoichiometry from the redox steps. Subsequent steps (e.g., Step B) are likewise expected to involve further oxidative activation, accompanied by the removal of at least 2H^+^/2e^–^, leading to reaction intermediates such as NH_2_OOH. Release of NO_2_^–^ is anticipated upon additional deprotonation and oxidation, whereas successive oxidative steps may yield NO_3_^–^ (Step D).

**Scheme 2:**
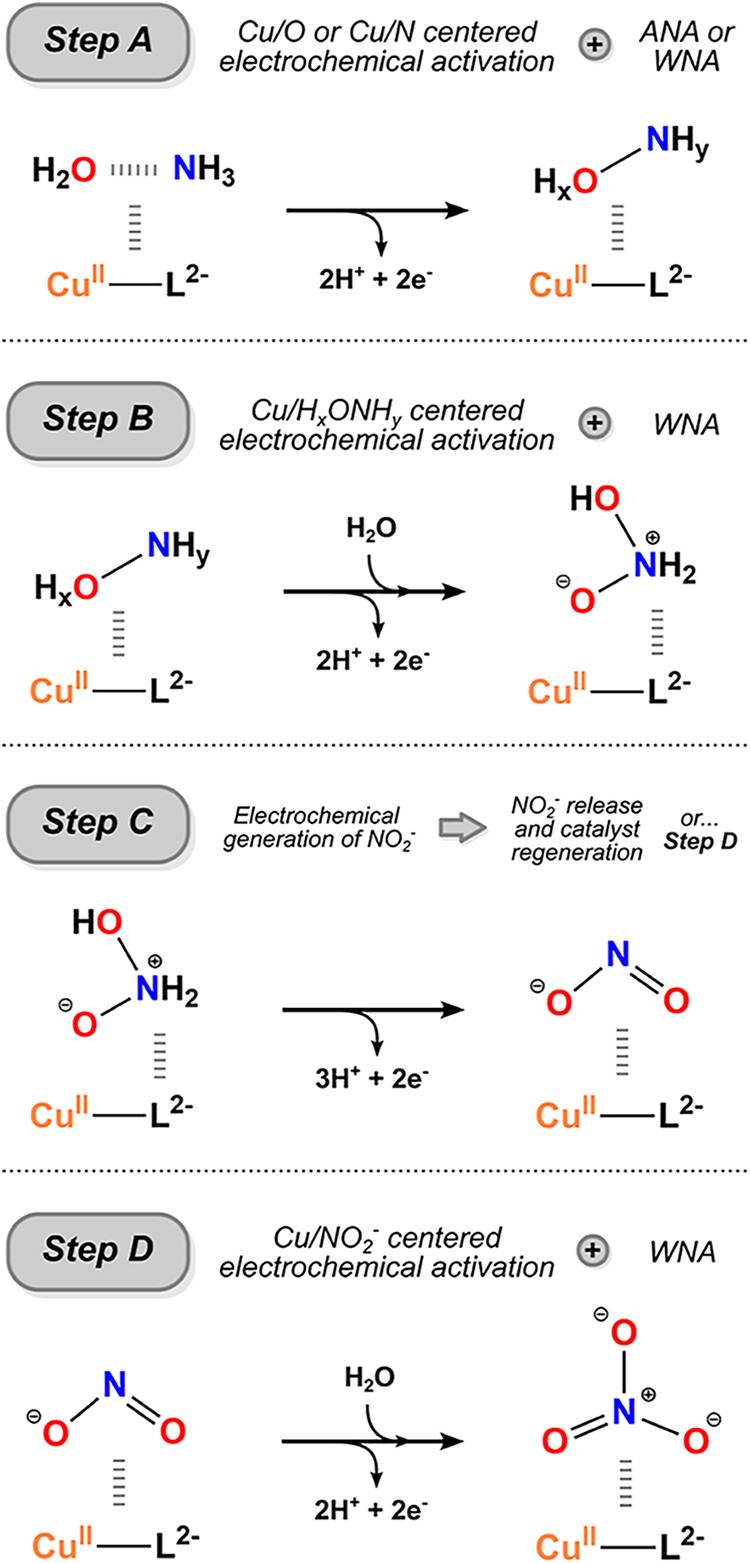
Proposed Stepwise Mechanism for Copper-Catalyzed AO to NO_*x*_ Species

It is worth emphasizing that the detailed mechanistic sequence of each transformation is considerably more intricate than depicted in Scheme 2, involving multiple reaction intermediates and transition states. Herein, we investigate the elementary reaction network of electrocatalytic ammonia oxidation, providing kinetic and thermodynamic insights into pathway competition and a molecular-level explanation for selectivity toward NO_2_^–^ and NO_3_^–^ formation from NH_3_ in aqueous media. To this end, we propose a detailed quantum mechanical investigation based on density functional theory (DFT) to perform a mechanistic screening of the formation of oxidized nitrogen species catalyzed by the [Cu­(pyalk)_2_] complex in ammonia-buffered aqueous solution (pH = 9). Specifically, we intend to deliver a comprehensive theoretical description of the elementary steps involved in the electrocatalytic cycle, which have not yet been systematically explored. To clarify the sequence of events and address the key mechanistic questions, the analysis is divided into three general sections: (i) Formation of the First N–O Bond, (ii) Formation of the Second N–O Bond, and (iii) NO_2_^–^ Release and O_2_N–O Coupling.

## Results and Discussion

### Formation of the First N–O Bond

Rudshteyn et al.,^24^ utilizing experimental and theoretical approaches, reported that the WO catalysis proceeds through the *cis* isomer of Cu­(pyalk)_2_, which can be formed via isomerization from the thermodynamically more stable *trans* isomer. This assignment was further supported by the close agreement between the theoretical (*cis* isomer) and the experimental UV–vis spectra in aqueous solution. Guided by these insights, we performed a structural search on the *cis* configuration of Cu­(pyalk)_2_ to identify the most stable arrangements as an active catalyst by incorporating ammonia molecules in the reaction model. As highlighted in Brudvig’s work,^21^ there is an apparent saturation of NH_3_ that is likely governed by second-sphere effects involving ammonia and the catalyst, in which NH_3_ preferentially forms hydrogen bonds to the alkoxide donor of Cu­(pyalk)_2_. However, axial coordination of NH_3_ as donor ligand to Cu^II^ cannot be completely ruled out; therefore, this event was also computationally addressed in the current study. In this context, we evaluated a range of structural arrangements in which NH_3_ is either weakly coordinated to the metal center or adopts plausible hydrogen-bonding or/and van der Waals interactions. The lowest-energy structures feature two NH_3_ molecules positioned above and below the plane of the complex (referred to as ^**2**^ **0**), consistent with a second-sphere binding motif stabilized by hydrogen bonding (H_2_NH···O-pyalk_2_). Electron density analyses were performed using Multiwfn^30^ to locate bond critical points (BCPs) within the atoms-in-molecules (AIM) framework,^31^ and hydrogen-bond strengths were estimated from the binding energies (BE) using the empirical relation of Emamian et al.^32^ These interactions were computed to be weak to moderate strength, with an average interaction energies of Δ*E* ≈ −2.38 kcal/mol and an average electron density at the bond critical point (ρ­(*r*)) of ≈0.014 a.u (see Figure S1 for more details).

In this direction, the first N–O bond coupling was computationally investigated, revealing two distinct mechanistic pathways: water nucleophilic attack (WNA) and ammonia nucleophilic attack (ANA), illustrated in Figures 1 and 2, respectively. Specifically regarding the WNA pathway­(Figure 1A), the process is initiated by a single-electron transfer ^**2**^ **0** →^**1**^ **1** involving an electron that is delocalized over the copper center, the alkoxide, and the pyridine nitrogen, but predominantly localized on copper (as evidenced by the spin density depicted in Figure S2A), thus corresponding to the Cu^II^/Cu^III^ oxidation. This step requires a reversible potential of 1.13 V, which is in excellent agreement with the experimentally reported quasi-reversible value, and is exergonic under an applied potential of 1.4 V (Δ*G* = −6.18 kcal/mol) as reported by Brudvig’s work.^21^ Subsequently, the structure of the Cu complex was adjusted in relation to the position of both ammonia molecules of Cu­(pyalk)_2_. This second coordination sphere reorganization could, in principle, take place before the SET reaction. However, this ligand reorganization was computed to be endergonic (see Figure S3A), supporting the proposed sequence (SET → second sphere ammonia reorganization) as the most favorable reaction pathway.

**Figure 1:**
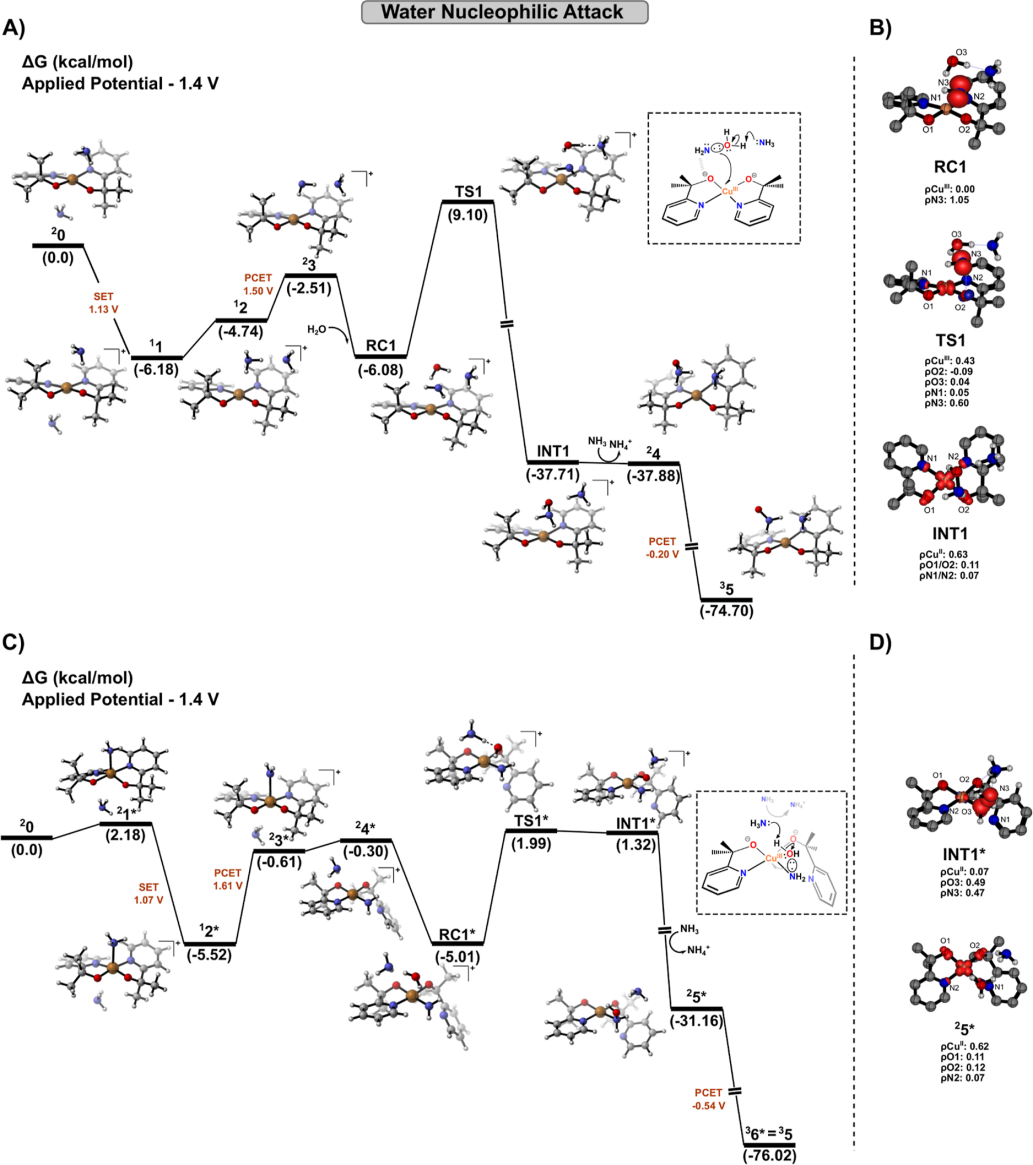
(A) Gibbs free energy profiles at 298.15 K for the water nucleophilic attack pathway occurring at the second-sphere of coordination, and (B) spin density analysis of key structures involved in the initial N–O bond formation via this route. (C) Gibbs free energy profiles at 298.15 K for the alternative axial site WNA pathway, and (D) its corresponding spin density analysis.

**Figure 2:**
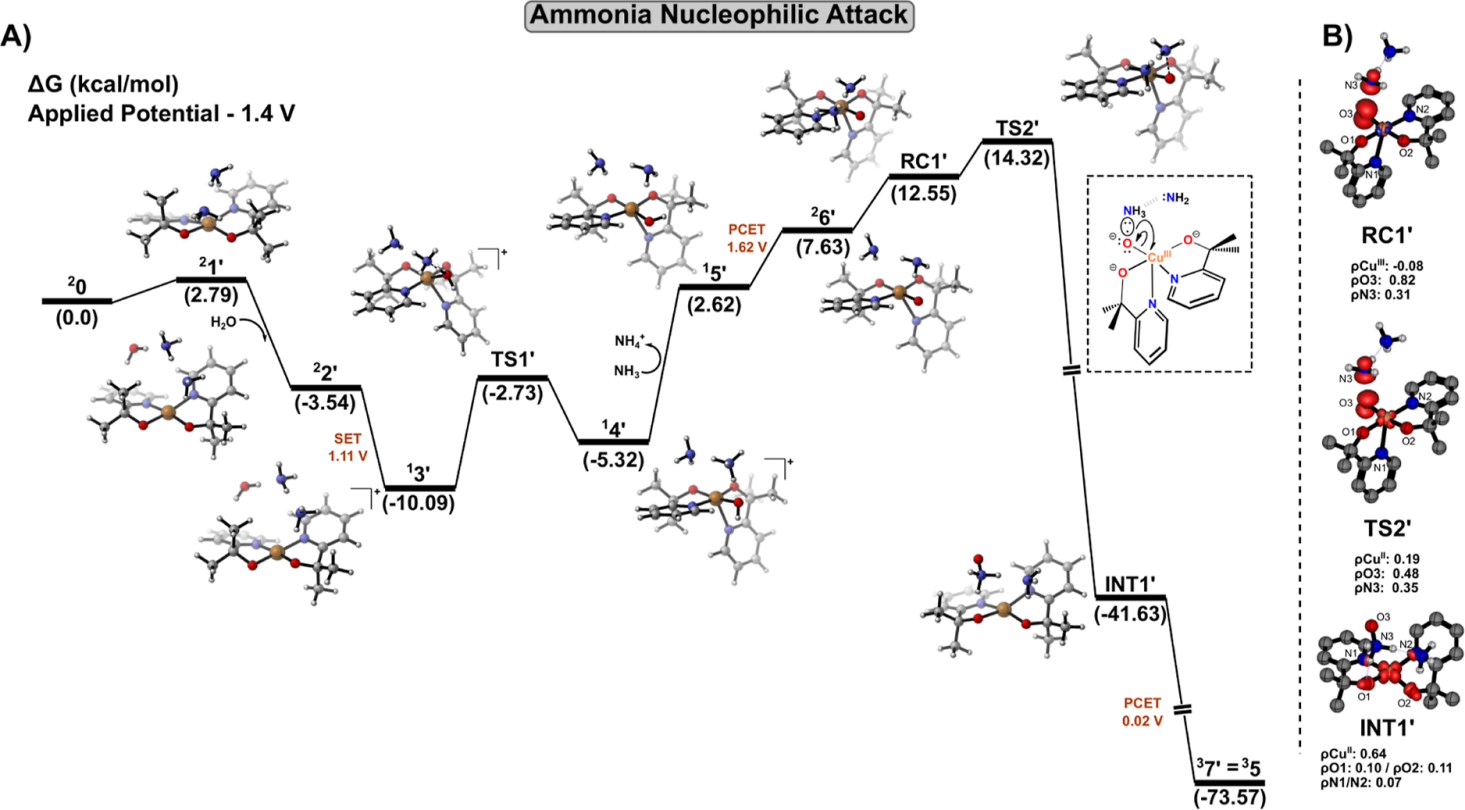
(A) Gibbs free energy profiles at 298.15 K and (B) spin density analysis of key structures involved in the formation of the first N–O bond via ammonia nucleophilic attack.

Subsequently, an additional electrochemical step occurs via a PCET event at 1.50 V involving ammonia, which creates an interaction with the alkoxide group of the ligand and the NH_2_ moiety, thereby converting ^**1**^ **2** to ^**2**^ **3** through a slightly endergonic process of 2.23 kcal/mol. This step maintains the +3 oxidation state of the metal center (ρ_Cu_ = 0.01). The quartet state was also tested, but the PCET event was estimated at 1.67 V, leading to a process 3.95 kcal/mol more energetic. At this stage, upon incorporation of an additional water molecule into the reaction model, the prereactive complex **RC1** was computed, containing an unpaired electron localized on the nitrogen atom of NH_2_ (its molecular structure is depicted in Figure 1B). The formation of **RC1** is responsible for the first σ-type N–O bond formation. This transformation proceeds through a concerted transition state (**TS1**), with a reaction barrier of 15.18 kcal/mol relative to **RC1**. This step involves proton transfer from water to NH_3_ via a two-center/three-electron (2c–3e) addition mechanism, facilitating an inner-sphere single-electron transfer (SET), as confirmed by IRC calculations (Figure S4A). Frontier molecular orbital (MO) analysis (Figure S4B) further supports this mechanism, revealing that the SOMO at **TS1** is delocalized over the N–O fragment *p*-orbitals and the Cu $d_{x^{2}-y^{2}}$ orbital, while the LUMO remains predominantly centered on the Cu $d_{x^{2}-y^{2}}$ orbital. This electronic configuration promotes a progressive increase in spin density at the copper center, consistent with an emerging +2 oxidation state (ρ_Cu_ = 0.43, Figure 1B). Subsequently, **INT1** is generated, exhibiting a computed spin density of approximately 0.63 at the copper center, a value characteristic of Cu^II^ complexes. This transformation can therefore be classified as a SET-WNA mechanism. Notably, our calculations indicate that ammonia significantly lowers the reaction barrier of the addition process. In this context, the proton transfer involving water is facilitated by the Brønsted–Lowry basicity of NH_3_. Indeed, we computed additional transition states both in the absence and in the presence of an extra ammonia molecule (Figure S3B), supporting its essential role as a proton acceptor during N–O bond formation. Interestingly, analogous buffer-mediated effects have been reported in other catalytic systems, such as phosphate-assisted WO catalysis.^26,27,33,34^

Finally, the proton is transferred to the medium via an NH_3_/NH_4_^+^ exchange, which is assumed to be diffusion-controlled and may readily occur due to the experimentally verified saturation of ammonia molecules around the complex. Moreover, at pH = 9, the concentrations of NH_3_ and NH_4_^+^ in solution are approximately equal. We further identify a highly exergonic process associated with a PCET event at −0.20 V, leading to the triplet species ^**3**^ **5**. Interestingly, the singlet analogue of ^**3**^ **5**, ^**1**^ **5**, undergoes a structural reorganization in which the NH_2_OH moiety adopts an equatorial orientation, forming a pentacoordinated complex with a distorted square-pyramidal geometry. In this case, the pyridine ligand migrates to the axial position. However, this singlet species is less stable by 24.8 kcal/mol and is therefore not expected to be mechanistically relevant to the electrocatalytic cycle.

Another WNA mechanism pathway for the initial N–O bond formation is highlighted in Figure 1C), where complex ^**2**^ **0** undergoes an initial rearrangement to structure ^**2**^ 1*. In this structure, one of the ammonia is coordinated at the axial site, corresponding to the **Cis-6** conformer in Figures S1. The process then proceeds via a SET at 1.07 V, which is consistent with the experimental value of 1.13 V. This is followed by a PCET event at 1.61 V, leading to the formation of ^**2**^ 3* species in an endergonic process (Δ*G* = 4.91 kcal/mol). The migration of the ligand from the axial to the equatorial site, coupled with the entry of a water molecule, stabilizes the system and results in the formation of RC1*. This reactive complex proceeds through **TS1**, a transition state characterized by a proton transfer from the water molecule to the NH_3_, yielding **INT1** with a low activation barrier of 7.00 kcal/mol.

In contrast to the concerted WNA typically observed in the secondary coordination sphere, the mechanism reported here is stepwise (nonconcerted). The subsequent exchange of NH_3_/NH_4_^+^ facilitates the direct formation of the N–O bond (25*), driving the metal complex to a formal +2 oxidation state (0.07 → 0.62). This step occurs in a barrierless manner, via 2c-3e interaction, a pathway supported by our relaxed scan (see Figure S4C). Finally, a subsequent PCET event at −0.54 V leads to ^**3**^ 6*. We also investigated the singlet state for this species; however, it was found to be significantly less stable, lying 29.31 kcal/mol higher in energy than the triplet state. It is also possible that a direct passage through a transition state occurs prior to the NH_3_/NH_4_^+^ exchange; however, despite extensive efforts, a saddle point could not be identified on this PES, suggesting a barrierless process once the required orientation is achieved. It is worth noting that multiple conformations may initially access this N–O bond formation via a WNA mechanism.

On the other hand, regarding the ANA pathway (Figure 2A), our calculations indicate a potential of 1.11 V, which was computed after a spatial second-sphere rearrangement of the ammonia molecules around the complex (i.e., the transformation from ^**2**^ **0** to the active catalytic species ^**2**^ **1′**). Analogous to the WNA mechanism, this reaction pathway was modeled by incorporating an additional water molecule, leading to ^**2**^ **2′**. Notably, the computed potential value is consistent with the SET process discussed above, thereby supporting the conclusion that the activation/oxidation step is critical and occurs predominantly at the metal center, due to the Cu spin density shift from ^**2**^ **2′** to ^**1**^ **3′** (0.63 → 0.00). Furthermore, our calculations indicate only a minor contribution from the ligand framework to the catalyst activation process. Thereafter, starting from ^**1**^ **3′**, a proton migration from the water molecule to ammonia via **TS1′** leads to the formation of complex ^**1**^ **4′** with an associated energy barrier of 7.36 kcal/mol. Following the proton transfer computed via **TS1′**, IRC calculations confirmed the coordination of the hydroxyl group to the copper center. Subsequently, a ligand rearrangement takes place through a concerted process involving either ^**1**^ **3′** and ^**1**^ **4′**. These reaction intermediates were computed to correspond to the reaction intermediates for **TS1′**. In this context, **TS1′** is specifically associated with the formation of a complex in which the hydroxyl ligand adopts an equatorial coordination position (^**1**^ **4′**). Notably, the reaction barrier discussed here (7.36 kcal/mol) is in good agreement with a previously reported theoretical study by Rudshteyn et al.,^24^ in which hydroxide coordination to the Cu^III^-(pyalk)_2_ complex adopts similar ligand rearrangement (^**1**^ **3′** →^**1**^ **4′**) before O–O bond formation in WO catalysis. In that case, the reaction barrier was computed to be approximately 6 kcal/mol, in close analogy to the N–O bond formation process described herein.

Afterward, the ammonium cation is replaced by an ammonia molecule in a manner analogous to that discussed for the SET-WNA mechanism, thereby restoring the neutral charge of the system (^**1**^ **5′**). Subsequently, a PCET event at 1.62 V, originating from OH^–^ as ligand in the coordination sphere, is therefore associated with the formation of Cu^III^–oxyl species (^**2**^ **6′**). Indeed, spin density analysis reveals one unpaired electron localized on the equatorial oxygen atom in ^**2**^ **6′** (ρ_O_ = 1.04), whereas copper remains in *d* ^8^ configuration (ρ_Cu_ = −0.04). Notably, the involvement of Cu^III^–oxyl species as reaction intermediates was previously hypothesized in Brudvig’s work to rationalize the competition between water and ammonia oxidation as a function of pH. Specifically, under relatively low pH conditions (pH = 9), the reduced availability of OH^–^ (compared to pH = 13) disfavors O–O bond formation, thereby shifting the chemoselectivity toward N–O bond formation via an ANA mechanism. As depicted in Figure 2A, a crucial feature promoting AO is a slight spatial rearrangement of the ammonia pair within the prereactive complex **RC1′**, in which one of the second-sphere ammonia molecules interacts with the oxyl radical formed as a ligand upon the Cu^III^ center. This interaction facilitates N–O bond formation through a two-center/three-electron (2c–3e) configuration (see details in Figure 2B). The N–O bond is finally formed via a nucleophilic attack of ammonia through **TS2′**. This step proceeds via a SET-ANA mechanism, in which a single-electron transfer from the three-electron bond to the metal center reduces its oxidation state from +3 to +2 Figure 2B). The corresponding free energy barrier was computed to be 6.69 kcal/mol relative to ^**2**^ **6′**, reflecting both the energetic cost of ammonia rearrangement and the subsequent energy release associated with N–O bond formation. The quartet surface was also explored, where ^**4**^ **6′** can be generated at the same potential (1.62 V). However, despite extensive efforts, a quartet transition state could not be located; this suggests that N–O bond formation proceeds preferentially through the doublet manifold.

It is worth highlighting the role of the assisting ammonia molecule, which acts in a manner distinct from that of a proton-accepting species. Instead, ammonia promotes charge transfer and polarization through electron-density donation via electron pair, thus providing significant stabilization to the complex, as indicated by calculations without this species (Figures S5 and S6). This effect is evident both in the conversion of **RC1′** as the precursor species and immediately after formation of the ONH_3_–Cu^II^ intermediate. Specifically, once the ONH_3_ fragment becomes uncoordinated, it migrates to the axial position, thereby interacting with the alkoxide group of the ligand. Concomitantly, the Cu^II^ complex restores its original square-planar geometry, yielding **INT1′**. Our calculations predict this transformation to be markedly exothermic, reflecting the substantial stabilization associated with formation of the new N–O σ-bond. Subsequently, a PCET event at 0.02 V leads to the triplet species ^**3**^ **7′**, which corresponds exactly to the reaction intermediate ^**3**^ **5** formed along the WNA pathway, with the ONH_3_ fragment adopting the same configuration. By contrast, the singlet analogue, ^**1**^ **7′**, is less stable by 26.0 kcal/mol and features the ONH_3_ moiety in an equatorial position within a distorted square pyramidal coordination geometry. However, similar to ^**1**^ **5**, this singlet species is not expected to be mechanistically relevant for the ANA pathway. Accordingly, the subsequent section of the manuscript proceeds from these unified stationary points on the free energy profile, namely ^**3**^ **7′** and ^**3**^ **5** at the triplet multiplicity (see Figure 2A).

### Formation of the Second N–O Bond

Our calculations reveal that the formation of the second N–O bond is initiated by an additional activation/oxidation step involving a SET computed at 0.31 V (Figure 3), which is associated with the redox conversion of the reaction intermediate ^**3**^ **5** in the triplet state to the Cu^II^ complex ^**2**^ **6** in the doublet state. This electrochemical event proceeds in parallel with a proton relocation within the pyalk framework, driven by the enhanced anionic character localized at the oxygen site. These mechanistic features are conveniently elucidated through two-dimensional chemical structure representations of the relevant computed reaction intermediates, as depicted in Figure S7A. Figure S7A is further supported by spin density analyses, which show that the single electron transfer originates predominantly from the NH_2_O fragment; thus, the copper center remains in the Cu^II^ oxidation state throughout the process, while the unpaired electron initially localized on NH_2_O, in which is later removed during the SET event, as subsequently depicted in Figure S7B.

**Figure 3:**
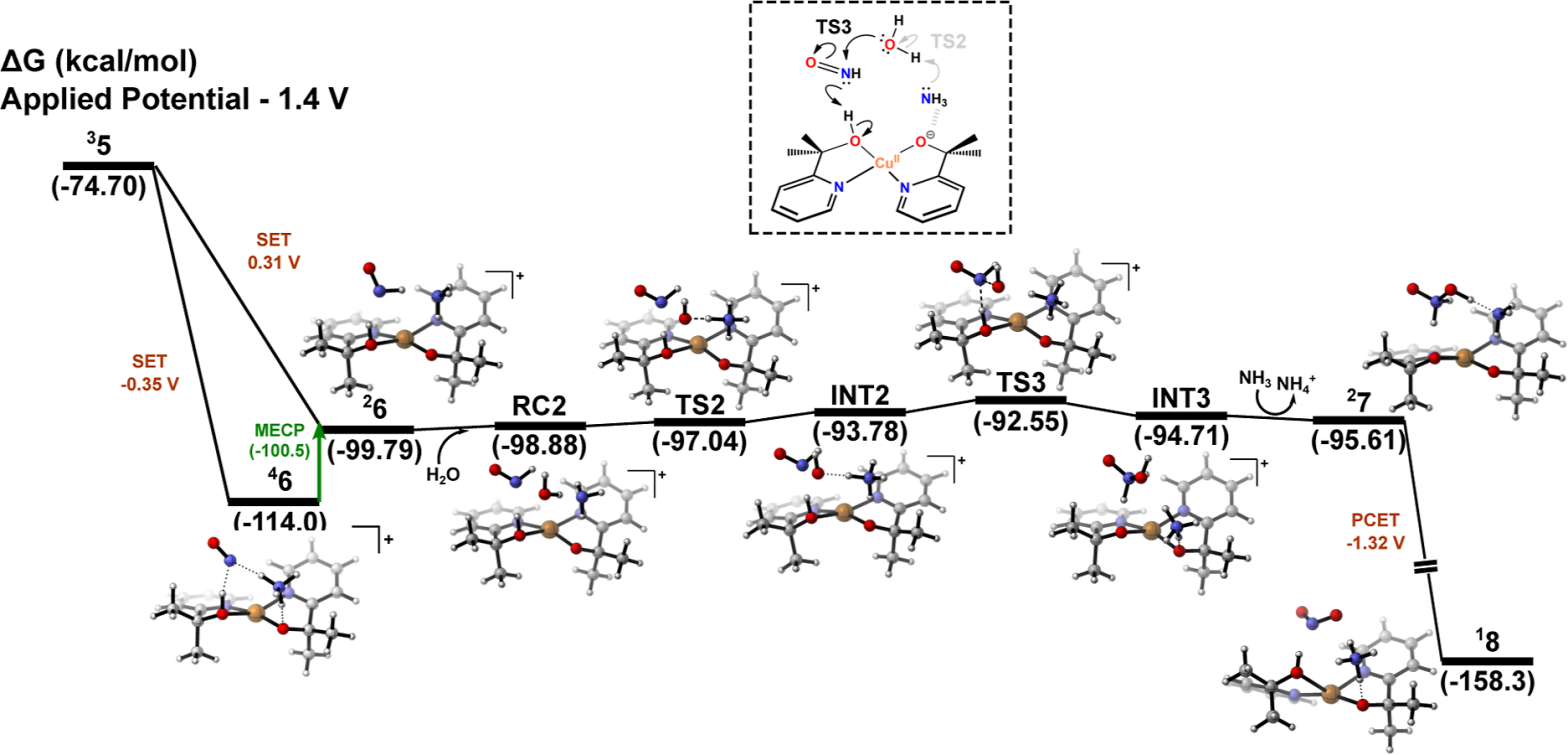
Gibbs free energy profiles at 298.15 K for formation of the second N–O bond.

In addition to ^**2**^ **6** as the precursor species, we identified an alternative reaction intermediate, denoted herein as ^**2**^ **6″**, which is alternatively accessed via a higher-potential SET event computed at 1.17 V. In contrast to ^**2**^ **6**, this process occurs without protonation of the pyalk alkoxide ligand, resulting in a species that is less stable by approximately 20 kcal/mol. To further clarify the electronic configuration involved in this redox process, we addressed a molecular orbital (MO) analysis of ^**3**^ **5**. Our calculations indicate that the higher oxidation potential arises from a metal-centered electron-transfer event involving the removal of an electron from a copper-based orbital (the α-SOMO-1 of ^**3**^ **5**; see the MO diagram in Figure S8). This orbital lies lower in energy than the NH_2_O-localized orbital corresponding to the α-SOMO associated with the formation of ^**2**^ **6**. Although we attempted to connect these two reaction intermediates through a transition state, no viable reaction pathway could be identified. Such an interconversion would be electronically demanding, likely involving an inner- or second-sphere SET process that engages both the Cu^II^ center and the NH_2_O moiety. We therefore conclude that ^**2**^ **6** and ^**2**^ **6″** arise from distinct reaction channels, with formation of the NH_2_O-centered species ^**2**^ **6** being favored under the experimentally relevant conditions modeled herein (aqueous media, pH, and temperature). In addition, we investigated a quartet potential energy surface (PES) starting from ^**4**^ **6**, although the N–O coupling seems improbable due to the repulsive behavior between NO and H_2_O (see Figure S9). We also explored alternative mechanistic scenarios beyond the electrochemical activation steps discussed above. In particular, we examined the triplet PES using different guess structures (see Figure S10). However, despite this extensive exploration, we were unable to locate stationary points capable of sustaining new productive reaction pathways. Furthermore, the products accessed along these routes are strongly endergonic, indicating that these computed pathways are mechanistically inaccessible.

Turning our attention to the reaction profile depicted in Figure 3, the incorporation of a water molecule into the second coordination sphere of the reaction model allows localization of the prereactive complex **RC2**, which is stabilized mainly by hydrogen-bonding interactions. **RC2** was computed to lie at −98.88 kcal/mol on the Gibbs free energy surface (relative to the catalyst and reactants). The formation of **RC2** from ^**2**^ **6** occurs on a very flat energy surface, thus supporting the smooth formation of **RC2**. Subsequently, a proton migration is mediated by transition state **TS2** (ν = −495.5*i* cm^–1^), which directly connects to intermediate **INT2**. Although **INT2** appears slightly higher in Gibbs free energy than the preceding transition state, this profile is a direct consequence of the PES topology in this region. To further validate this reaction channel, a relaxed scan was performed along the reaction coordinate (Figure S11A), confirming **TS2** as a well-defined electronic energy maximum, with **INT2** residing in a subsequent local minimum. The apparent inversion in the free energy profile arises from the inclusion of vibrational and thermal corrections. In such flexible transition metal complexes, low-frequency vibrational modes associated with weak intermolecular interactions and soft degrees of freedom can disproportionately stabilize transition states relative to intermediates. This phenomenon has been observed in other transition metal complex systems^35,36^ and does not compromise the mechanistic interpretation, as the underlying electronic potential energy surface maintains the expected sequence of stationary points.

After formation of **INT2**, the second N–O bond is finally established via a direct nucleophilic attack of the in situ-generated hydroxide through transition state **TS3**, leading to complex **INT3** as the reaction product. The step-by-step mechanism for N–O bond formation via **TS2** and **TS3** is evidenced by the displacement vectors in Figure S11B. Although this reaction step could be classified under a classical WNA-type mechanism, our calculations reveal that it is more accurately described as a hydroxide nucleophilic attack (HNA), given the nonconcerted nature of the two processes (proton abstraction and N–O bond formation), in marked contrast to the first WNA step shown in Figure 1. Notably, atomic spin density analyses reveal no change in the oxidation state of the copper center during the computed HNA mechanism (Figure 4), which is fully consistent with a closed-shell nucleophilic process. Indeed, the hydroxide ion donates its lone electron pair to interact with an antibonding π* orbital of the ONH fragment. In concert, this transformation is accompanied by proton migration between the alkoxide ligand and the HNOOH moiety, reflecting the highly dynamic Lewis acid–base interactions that facilitate charge redistribution within the Cu complex and explain the stabilization of the structures shown in Figure 3.

**Figure 4:**
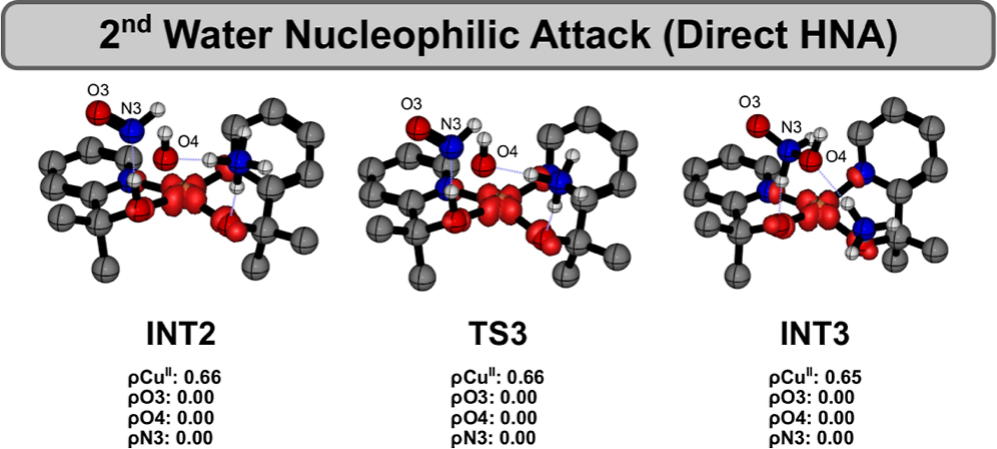
Spin density of key structures involved in second N–O bond formation.

Likewise to the first N–O bond formation, we propose that ammonia saturation and the applied buffer facilitate this process through proton exchange between ammonia and the ammonium ion, thereby promoting proton migration to the reaction medium. In this context, **INT3** evolves into the doublet species ^**2**^ **7**, which experiences a modest stabilization, likely due to the presence of a hydrogen bond between the hydroxyl oxygen of the NH_2_OOH moiety and the nitrogen atom of ammonia. Subsequently, a PCET event with a computed potential of −1.32 V was identified, involving electron removal from the HOMO of the NH_2_OOH fragment, as shown in Figure S12. This redox process is strongly exergonic, lowering the Gibbs free energy by 62.69 kcal/mol. The redox event is followed by sequential proton-transfer steps: one from the ammonium group of NH_2_OOH to the alkoxide ligand of pyalk, and another one from the hydroxyl oxygen of NH_2_OOH to a neighboring ammonia molecule. Concomitantly, an electron is transferred to the metal center, reducing the copper oxidation state to +1. To support this mechanistic picture, natural bond orbital (NBO) analysis was performed, revealing that the electronic configuration of the metal center in the singlet species ^**1**^ **8** corresponds to a *d* ^10^ manifold. In addition, a qualitative Mulliken atomic charge population analysis indicates that the positive character of the copper center decreases from *q*(Cu) = +0.396 to *q*(Cu) = +0.167, thus supporting the Cu^I^ complex formation. In addition, to further expand our mechanistic analysis, we also investigated the triplet species ^**3**^ **8**, generated without proton migration. However, this species was computed to be significantly higher in energy, thereby ruling out mechanistic scenarios in which the copper center remains in the +2 oxidation state (see Figure S13).

Complementarily, we also identified an alternative reaction pathway analogous to that described throughout Figure 3, in which the water molecule approaches the metal complex with a different orientation. This pathway is illustrated in Figure S14 of the Supporting Information. Although both reaction paths share identical stationary points, a key difference was identified for the complex ^**3**^ **8″**. As a consequence, the PCET associated with the final step of the free energy profile shown in Figure S14 was estimated to occur at −0.42 V and is therefore highly exergonic, albeit significantly less favorable than the corresponding process (^**2**^ **7** →^**1**^ **8**) depicted in Figure 3, differing by approximately 23 kcal/mol. Notably, no proton migration to the alkoxide ligand is observed along the pathway corresponding to Figure S14, as illustrated by the two-dimensional schematic shown in Figure S15. Accordingly, we propose that the reaction surface discussed in the main text is both kinetically and thermodynamically preferred, whereas species such as ^**3**^ **8″** are less stable, may arise transiently, and can access a more stable form through surface crossing. These findings highlight the intricate mechanistic landscape for the first and second N–O bond formations revealed by our calculations.

### NO_2_^–^ Release and O_2_N–O Coupling

By scrutinizing the complex ^**1**^ **8**, it is evident that the nitrite anion has already been formed. In this context, this species can dissociate from the coordination sphere as an ion pair together with the ammonium cation, being subsequently replaced by two ammonia molecules occupying alternate axial positions, yielding ^**1**^ **9″** as depicted in Figure 5. This exchange process was computed as energetically demanding with a free energy cost of 12.60 kcal/mol, whereas NO_2_^–^ remains associated with the complex through intermolecular interactions, specifically involving the pyalk moiety of the ligand, in agreement with experimental insights.^21^ Subsequently, a PCET step was computed with a potential of −0.68 V, in which the ligand is deprotonated while the metal center is oxidized from Cu^I^ to Cu^II^. At this stage, the active catalyst complex ^**2**^ **0** is regenerated, so the catalytic cycle restarts. Interestingly, our calculations also indicate a second, and more plausible, reaction route for NO_2_^–^ release that follows an inverse sequence, in which the PCET precedes the ligand exchange step. In this alternative pathway, an exergonic PCET of 0.00 V leads to species ^**2**^ **9**, where the NH_4_^+^ cation in ^**1**^ **8** is deprotonated instead of the ligand. Subsequently, the exchange of the nitrite anion with one ammonia molecule at the opposite axial face of the complex results in a slight stabilization of 2.50 kcal/mol, forming ^**2**^ **10′**. Finally, deprotonation of the ligand regenerates the active catalyst ^**2**^ **0**.

**Figure 5:**
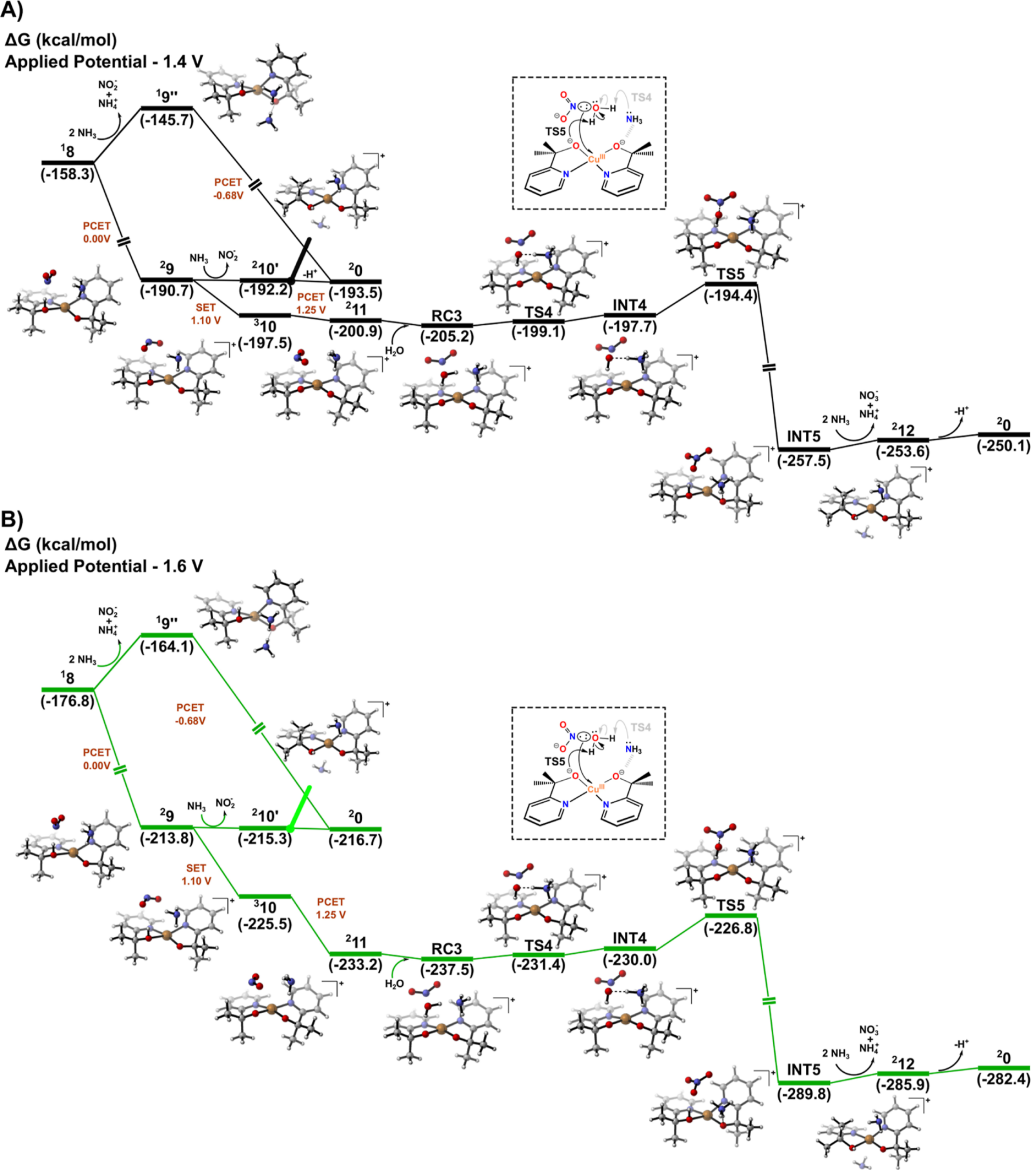
Gibbs free energy profiles at 298.15 K for formation of the third N–O bond: (A) applied potential of 1.40 V and (B) applied potential of 1.60 V.

The calculations also provide insights into the competition between NO_2_^–^ release and the formation of NO_3_^–^ derived from intermediate ^**2**^ **9**. In Brudvig’s experiments,^21^ the authors observed a potential-dependent selectivity between NO_2_^–^ and NO_3_^–^ formation over an applied potential range of 1.40–1.60 V. Specifically, potentials in the range of 1.50–1.60 V favor NO_3_^–^ formation, whereas potentials below 1.50 V favor nitrite formation (62% vs 25%). In light of these observations, we investigated the possible origins of this electroselectivity. First, based on the preceding N–O bond formation steps, we identified that an additional electrochemical activation is likely required to promote the O_2_N–O coupling. From this perspective, we propose a redox sequence involving NO_2_^–^, consisting of an initial SET (1.10 V) followed by a PCET (1.25 V), in which the metal center is oxidized (Cu^II^ → Cu^III^) and the ligand is deprotonated, leading to ^**2**^ **11** as the resulting complex. Regarding the SET process, spin density analysis of ^**3**^ **10** indicates that the removed electron originates from the nitrogen lone pair of NO_2_^–^. This electronic configuration is further supported by the Frontier molecular orbital energy diagrams of ^**2**^ **9** (Figure S16), which show a nonbonding orbital of predominantly NO_2_^–^ character at higher energy relative to the copper centered d-type orbital. Conversely, the relative instability of the singlet species ^**1**^ **10**, computed to be 16.31 kcal/mol higher in energy (wherein NO_2_^–^ interacts with a Cu^III^ center), demonstrates that oxidation of Cu^II^ as the first redox step is not energetically favorable (see Figure S17). We also explored alternative electrochemical redox sequences, such as PCET followed by SET or two consecutive SET events. However, the intermediates formed through these pathways were computed to be highly endergonic and significantly less stable than those obtained via the redox sequence proposed in Figure 5. In addition, we evaluated the possibility of direct coordination of NO_2_^–^ to the copper center. The resulting pentacoordinated complex was found to be substantially higher in energy, thereby ruling out occupation of an equatorial site by the NO_2_^–^ anion in the first coordination sphere (see Figure S18 for details).

By comparing the energy profiles computed at the applied potentials of 1.40 V (Figure 5A) and 1.60 V (Figure 5B), it becomes possible to rationalize the NO_2_^–^/NO_3_^–^ inversion through the pronounced stabilization at higher applied potentials—in this case 1.60 V (Figure 5B)—associated with the proposed electrochemical events. Specifically, the energy required for the O_2_N–O coupling step (**TS5**) is considerably lower than that associated with catalyst regeneration following NO_2_^–^ release from the system (^**2**^ **9** →^**2**^ **0**); as a consequence, NO_3_^–^ formation becomes energetically more favorable. In contrast, under lower applied potentials, the stabilization provided by the oxidative redox steps, including the energy barrier associated with NO_3_^–^ formation, is not energetically competitive with the direct release of NO_2_^–^ into solution from the vicinity of the ligand. Overall, we propose that the electrochemical activation of NO_2_^–^ within the coordination environment of the complex is the primary origin of the experimentally observed inverse selectivity between NO_2_^–^ and NO_3_^–^. From this perspective, the higher amounts of NO_3_^–^ produced during the electrocatalytic ammonia oxidation with [Cu­(bipyalk)]^+^ (bipyalk = 2-[(2,2′-bipyridin)-6-yl]­propan-2-oxide), for example, may be associated with lower oxidation potentials for NO_2_^–^ or reduced energy barriers for the O_2_N–O coupling step, thereby rendering this pathway selectively dominant. Furthermore, this ligand is tridentate, leaving one equatorial coordination site available for NO_2_^–^ binding in a square-planar geometry. As a result, NO_2_^–^ release into solution via decoordination from the copper center is expected to be less favorable compared to the catalysis mediated by the pyalk system proposed herein, in which NO_2_^–^ preferentially interacts with the ligand framework through intermolecular interactions rather than occupying the first coordination sphere.

The mechanism of the third N–O bond formation was computed to be very similar fashion to that previously proposed herein. First, the inclusion of an explicit water molecule in the system leads to the formation of **RC3**, accompanied by a slight stabilization of the complex. Once again, a proton transfer to the assisting ammonia molecule was identified through **TS4** (ν = −365*i* cm^–1^) on a flat PES, yielding **INT4** as the product, which appears slightly higher in Gibbs free energy than the transition state upon the inclusion of vibrational and thermal corrections, as discussed previously. In this intermediate, the newly formed hydroxide interacts with the anion radical NO_2_^–^ through a two-center/three-electron (2c–3e) bond, as confirmed by spin density analysis (Figure 6). Subsequently, the O_2_N–O coupling proceeds via **TS5**, which lies 3.2 kcal/mol above **INT4** in energy. IRC calculations confirm **INT5** as the product of this step, which involves a concerted proton transfer from the attacking hydroxide to the pyalk ligand. Accordingly, analysis of the atomic spin density values shown in Figure 6 indicates that the N–O bond formation mechanism involves a SET from the (2c–3e) bond to the metal center, accompanied by a change in the copper oxidation state from +3 to +2, as further illustrated by the two-dimensional structural representations. Finally, the ion pair NO_3_^–^···NH_4_^+^ dissociates from the coordination sphere and is replaced by two ammonia molecules through a ligand-exchange process, yielding ^**2**^ **12**. In the last stage of the catalytic cycle, an additional deprotonation of the ligand restores the original catalyst. Overall, the complete catalytic cycle encompassing both nitrite and nitrate formation pathways is summarized in Figure 7. Our results show the oxidation-state shift of copper as II (^**2**^ **0**) → III (^**1**^ **1**/^**1**^ **3′**) → II (**INT1**/**INT1′**) → I (^**1**^ **8**) → II (^**2**^ **9**) → III (^**3**^ **10**) → II (**INT5**). In addition, the oxidationstate evolution of the nitrogen atom in ammonia, upon its conversion to NO_2_^–^ or NO_3_^–^, follows the sequence: −III (^**2**^ **0**) → −I (^**3**^ **5**) → I (^**2**^ **6**) → III (^**1**^ **8**) or V (^**2**^ **12**).

**Figure 6:**
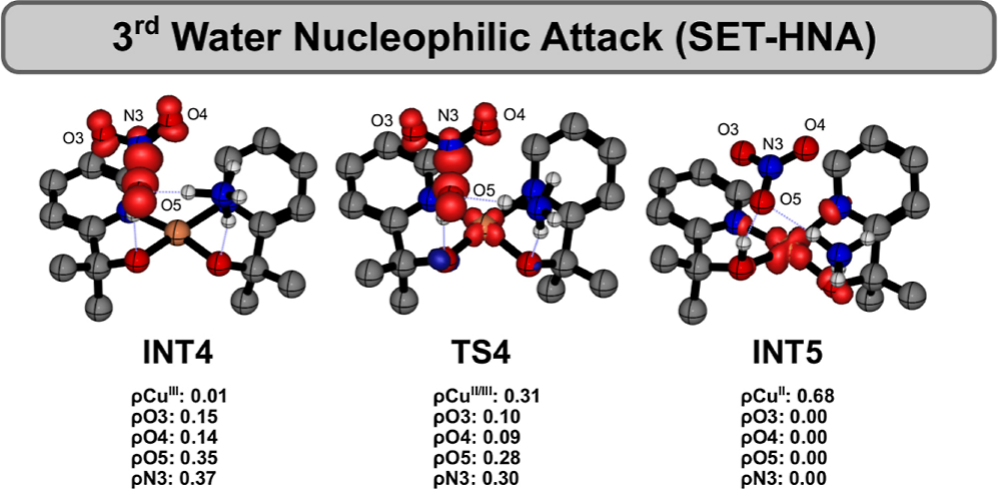
Spin density of key structures involved in third N–O bond formation.

**Figure 7:**
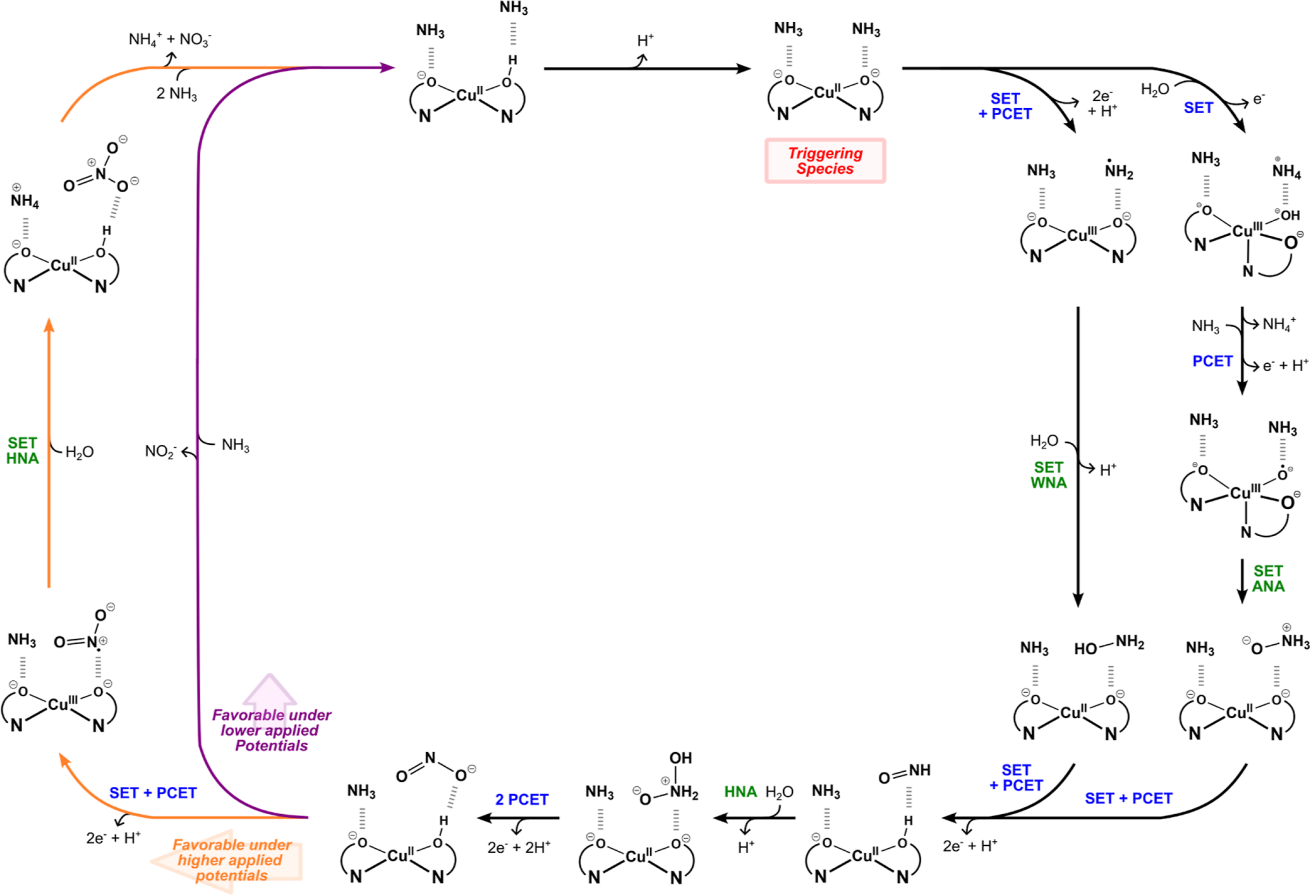
Proposed catalytic cycle for electrochemical ammonia oxidation catalyzed by the complex [Cu­(pyalk)_2_].

## Summary and Conclusions

In summary, the present theoretical work computationally investigates the mechanisms of electrochemical ammonia oxidation to nitrite and nitrate in aqueous media catalyzed by the [Cu­(pyalk)_2_] complex (at pH = 9). Despite recent advances in terms of the catalytic efficiency for the electrochemical ammonia oxidation, several mechanistic aspects have remained unclear. In this context, our results aimed to clarify key mechanistic issues—including the origin of N–O bond formation and the identity of the selective-determining step—and shed light on the inversion between NO_2_^–^ and NO_3_^–^ under the influence of the applied potential. We demonstrate that electrochemical activation of the catalyst and/or the substrate serves as the driving force for all three N–O bond-forming events to take place. Specifically, we propose that the first N–O bond formation proceeds via two distinct mechanisms, namely single electron transfer-ammonia nucleophilic attack (SET-ANA) or single electron transfer-water nucleophilic attack (SET-WNA). Our calculations indicate that these mechanistic scenarios represent the major kinetic bottleneck of the catalytic cycle. Subsequently, NO_2_^–^ formation occurs through a direct hydroxide nucleophilic attack (HNA) mechanism, for which a very low reaction barrier was computed. Furthermore, we propose that the subsequent electrochemical activation/oxidation steps act as the key driving forces governing the experimentally observed selectivity, wherein NO_3_^–^ is predominantly formed under higher applied potentials, as it results in increased stability, which in turn favors the pathway toward NO_3_^–^ production. In contrast, under lower applied potentials, the energy barrier associated with NO_3_^–^ formation is no longer energetically competitive with the direct release of NO_2_^–^ into solution from the vicinity of the ligand, thus explaining the preferential formation of NO_2_^–^. Finally, the third N–O bond formation proceeds through a SET-HNA pathway with feasible computed reaction barriers.

Overall, the involvement of ammonia molecules in the second coordination sphere of the Cu complexes, supported by the presence of a buffered solution, plays a crucial role throughout the catalytic cycle. In particular, ammonia molecules enhance the nucleophilic character of the reactive species, thereby facilitating N–O bond formation. Indeed, our calculations show that the catalytic activity of [Cu­(pyalk)_2_] arises not only from its electrophilic site (the metal center) but also from nucleophilic sites within the ligand framework, specifically, on the anionic alkoxide groups. These electrophilic and nucleophilic regions in the first and second sphere of the coordination complexes promote stabilizing intermolecular interactions, such as hydrogen bonding, which are essential for enabling energetically viable N–O coupling processes in aqueous solution. In conclusion, our investigation provides computational insights into the electrochemical ammonia oxidation cycle in aqueous solution, particularly at the molecular mechanistic level, highlighting key kinetic and thermodynamic factors that might be relevant to the rational design of Cu-based catalysts. It is important to emphasize that, given the complexity of the catalytic system and the inherent limitations of the molecular modeling approach, alternative catalytic pathways cannot be ruled out. New studies are currently ongoing to explore these possibilities and will be reported in due course.

## Computational Methods

The quantum mechanical investigation proposed herein is based on DFT electronic structure calculations carried out using the Gaussian 09 software package.^37^ Geometry optimizations and vibrational frequency calculations for all stationary points were performed using the B3LYP^38^ density functional with Grimme’s D3 dispersion correction.^39^ The def2-SVP^40^ basis set was employed for C, N, and H atoms, whereas O atoms in the pyalk ligands bearing a localized negative charge were treated with a specific diffuse basis set (6–31 + G­(d)),^41,42^ as recommended by Rudshteyn and co-workers.^24^ The Cu center was described using the LANL2DZ­(f) basis set,^43,44^ which is associated with an effective core potential for the core electrons. The solvation effects were included directly in the free energies by employing the SMD continuum solvation model with the default parameters for water as the solvent during geometry optimizations and vibrational frequency calculations.^45^ This electronic structure methodology was further validated through benchmarking (see Table S1), in which the experimental redox potential associated with the Cu^II^/Cu^III^ couple (1.13 V vs NHE; 1.66 V vs RHE)^21^ was well reproduced. Furthermore, this level of theory also showed excellent agreement between the optimized gas-phase geometry and the reported crystallographic structure (see details in Table S2), thus providing reliable energetic and structural insights. All transition states reported in the present study were confirmed by the presence of a single imaginary frequency, whereas minima exhibited only positive vibrational frequencies. In selected cases, transition states were located using the synchronous transit-guided quasi-Newton methods (QST2 and QST3).^46^ Multiple electronic states were examined by varying the total spin multiplicity. Intrinsic reaction coordinate (IRC)^47,48^ calculations were performed to verify the connectivity of each transition state to the corresponding reactant and product along the reaction pathway. Moreover, minimum energy crossing points (MECP) were calculated trough Harvey’s algorithm, as implemented in EasyMECP code.^49^ Gibbs free energies in aqueous solution were computed at 1 M and room temperature using the GoodVibes code,^50^ applying quasi-rigid-rotor-harmonic-oscillator corrections with a cutoff of 100 cm^–1^ (Grimme method as default).^51^ Electrochemical potentials for the transformations along the AO catalytic cycle were computed at pH = 9 using the same formalism previously proposed by Truhlar and co-workers,^52^ providing excellent agreement with experimental values, as previously demonstrated by our group for Cu-based water oxidation (WO) catalysis.^27,33^ Standard redox potentials (*E*°) were referenced to the Standard Hydrogen Electrode (SHE) with pH dependence accounted for via the Nernst equation (assuming a 1e^–^/1H^+^ stoichiometry). For steps under an applied potential (*U* = 1.4 or 1.6 V vs SHE), the Gibbs free energy change (Δ*G*) was adjusted accordingly (for more details, see Section S1 of the Supporting Information).

## Supplementary Material









## Notes

Additional figures regarding the conformational analysis and reaction mechanism (PDF)
A file containing all Cartesian coordinates of the main stationary points reported (XYZ)
First_N–O_bond.xyz (XYZ)
Second_N–O_bond.xyz (XYZ)
