# Supplementary material for: Origins of Nitrite and Nitrate Selectivity in Aqueous Electrocatalytic Ammonia Oxidation by a Mononuclear Copper Catalyst
Source: Inorg Chem. 2026 May 18;65(21):11794–806. doi: 10.1021/acs.inorgchem.6c00859 (PMC13231411; doi:10.1021/acs.inorgchem.6c00859)
Supplement: Supplementary file 1 [file ic6c00859_si_003.pdf]

Supporting Information

Origins of Nitrite and Nitrate Selectivity in Aqueous Electrocatalytic Ammonia Oxidation by a Mononuclear Copper Catalyst

Joel Leitão Nascimento[a], João Pedro C. S. Neves[a], Roberto Rivelino[b], Vitor H. Menezes da Silva[a,*] and Tiago Vinicius Alves[a,*]

[a]*Departamento de Físico-Química, Instituto de Química, Universidade Federal da Bahia, Rua Barão de Jeremoabo, 147, Salvador, Bahia, 40170-115, Brazil*
[b]*Departamento de Físico-Química, Instituto de Química, Universidade Federal da Bahia, Rua Barão de Jeremoabo, Salvador, Bahia 40170-115, Brazil*

*E-mail address: vhugo@iq.usp.br and tiagova@ufba.br

Tables

S1 DFT benchmarking of SET potentials for the $^{2}\mathbf{0} \rightarrow {}^{1}\mathbf{1}$ transformation. . . . S6

S2 Geometrical parameters of the **trans** form of Cu(Pyalk)$_2$. Additional structural data are included for comparison purposes. S6

Figures

S1 Different ammonia coordination and organization modes in Cu(pyalk)$_2$ structures S7

S2 Spin density isosurface of species $^{2}\mathbf{0}$ and $^{2}\mathbf{1^*}$. S7

S3 Gibbs free energy profiles for alternative pathways: A) Ammonia reorganization prior to metal SET, B) transition states along the WNA Pathway, and C) initial water coordination in the ANA mechanism. S8

S4 A) Intrinsic reaction coordinate (IRC) profile for **TS1** connecting to **INT1**. B) Three-dimensional representation of molecular orbital of **TS1** transition state. C) A relaxed scan initiated from $^{2}\mathbf{5^*}$ for the N–O bond dissociation showing a barrierless process. S9

S5 Mulliken charge analysis for **RC2′** emphasizing the role of auxiliary ammonia. S9

S6 SET-ANA energy profiles with (in black) and without (in red) auxiliary ammonia. S10

S7 A) Two-dimensional representations and of the proposed transient species involved the transformation from species $^{3}\mathbf{5}$ to $^{2}\mathbf{6}/^{2}\mathbf{6''}$ and B) the corresponding spin densities distributions. S10

S8 Three-dimensional representation of molecular orbital of $^{3}\mathbf{5}$ specie. S11

S9 Possible quartet PES for the second N−O coupling. S11

S10 Gibbs free energy profiles for alternative pathways of the initial step leading to formation of the second N–O bond on the triplet surface. S12

S11 A) A relaxed scan initiated from TS2 connecting RC2 and INT2 shows a lower electronic energy barrier; B) displacement vectors for the imaginary vibrational mode of **TS2** and **TS3**. S12
S12 Three-dimensional representation of molecular orbital of ^{2}**7** specie. S13
S13 Two-dimensional representations of the proposed transient species involved the transformation from species ^{2}**7** to ^{3}**8**/^{1}**8**. S13
S14 Gibbs free energy profiles for alternative pathways for the formation of the second N–O bond, in which the water molecule approaches the metal complex from above rather than from the front. S14
S15 Molecular orbitals of $^{2}7''$, and two-dimensional representations of this proposed transient species involved in the transformation from $^{2}7''$ to $^{3}8''/^{1}8''$. S14
S16 Three-dimensional representation of molecular orbital of species ^{2}**9**. S15
S17 Two-dimensional representations of the proposed transient species involved the transformation from species ^{2}**9** to ^{3}**10**/^{1}**10**. S16
S18 Gibbs free energy profiles for alternative pathways of the initial step that leads to NO_2^- release, starting from the equatorial isomer: A) N-bound and B) O-bound coordination to the metal center. S17

S1 Gibbs Free Energies and Electrochemical Potential Calculations

To evaluate the thermodynamics of the electron transfer processes (SET or PCET) involved in the catalytic ammonia oxidation, the redox potentials (E°) were derived from the calculated Gibbs free energies. Under standard conditions (pH = 0), the E° for a given redox couple is defined as follows:

$$E^\circ_{\mathrm{O|R}} = \frac{G^\circ(\mathrm{O,aq}) + n^{\mathrm{H+}} G^\circ(\mathrm{H^+,aq}) - G^\circ(\mathrm{R,aq})}{n_e F} - E^\circ_{\mathrm{SHE}} \tag{1}$$

where $G^\circ(\mathrm{O,aq})$ and $G^\circ(\mathrm{R,aq})$ are the absolute standard Gibbs free energies of the oxidizing and reducing agents, respectively. The term $G^\circ(\mathrm{H^+,aq})$ corresponds to the free energy of the proton in solution, taken here as −270.3 kcal/mol [1]. E°_{SHE} stands for the absolute potential of the standard hydrogen electrode (SHE), set at 4.281 V [2], and F is the Faraday constant (23.08 kcal mol^{-1}V^{-1}). In the context of the single-electron transfer process, the $G^\circ(\mathrm{H^+,aq})$ term is omitted. As detailed in the Computational Methods section, the final Gibbs free energy corrections to 1 mol L^{-1}) in water solution at room temperature were computed using the GoodVibes code, considering a frequency cutoff of 100 cm^{-1}, and a correction for water concentration (55.56 mol L^{-1}) of 4.27 kcal/mol. The total Gibbs free energy was obtained directly from calculations using the SMD implicit solvation model [`SCRF(SMD, Solvent=Water)`], ensuring that solvation effects were consistently included in the potential energy surface.

To adjust the previously calculated standard redox potentials (E°) to the experimental conditions (pH = 9), the Nernst equation was employed:

$$E_{\mathrm{O|R}} = E^\circ_{\mathrm{O|R}} - \frac{n^{\mathrm{H+}}}{n_e}\frac{RT}{F}\ln(10)\mathrm{pH} \tag{2}$$

For a PCET process (1e$^-$/1H$^+$) at 298.15 K, the equation simplifies to a correction of approximately −0.532 V at pH = 9. Furthermore, to account for the externally applied potential (U = 1.4 V or 1.6 V vs. SHE) used in the experimental setup, the Gibbs free

energy change (ΔG) for each step is given by:

$$\Delta G = n_e F(E - U) \tag{3}$$

[1] M. D. Tissandier, K. A. Cowen, W. Y. Feng, E. Gundlach, M. H. Cohen, A. D. Earhart, J. V. Coe, T. R. Tuttle, J. Phys. Chem. A 1998, 102, 7787.

[2] J. E. Bartmess, J. Phys. Chem. 1994, 98, 6420.

S2 Benchmarking and Additional Information

Table S1: DFT benchmarking of SET potentials for the $^2\mathbf{0} \rightarrow {}^1\mathbf{1}$ transformation.

DFT	SET Potential [Cu(II)/Cu(III)] (V)	MUD (%)
**Multiplicity = Singlet**		
B3LYP-D3/BS1	1.10	3.0
WB97XD/BS1	1.49	32.3
M06/BS1	1.24	9.4
M06-2X/BS1	2.31	104.8
M06L/BS1	0.66	41.5
TPSSH	0.76	32.7
B3LYP-D3/BS2	1.05	7.5
M06/BS2	1.26	11.6
B3LYP-D3/BS3	1.13	0.2
M06/BS3	1.29	13.8
**Multiplicity = Triplet**		
B3LYP-D3/BS1	1.34	18.6
WB97XD/BS1	1.51	33.2
M06/BS1	—	—
M06-2X/BS1	1.97	74.5
M06L/BS1	0.96	15.3
TPSSH/BS1	1.02	10.0

[a] BS1 corresponds to a mixed basis set (def2-SVP and LANL2DZ(f))), where LANL2DZ(f) is applied to the copper metal (Cu).
[b] BS2 corresponds to the def2-SVP basis set.
[c] corresponds to a mixed basis set (def2-SVP,6-31+G(d), LANL2DZ(f)), where 6-31+G(d) is applied only to the oxygen atom (O) and LANL2DZ(f) to the copper metal (Cu).

Table S2: Geometrical parameters of the **trans** form of $Cu(Pyalk)_2$. Additional structural data are included for comparison purposes.

	Cu-N (Å)	Cu-O (Å)	N-Cu-O (intra) (°)	N-Cu-O (inter) (°)	N-C-C-O (°)
Rudshteyn et al.[a]	1.99	1.89	83.7	96.3	0
Fisher et al.[b]	1.972(2)	1.883(1)	84.04(5)	95.96	-6.528
This work	1.99	1.90	84.00	95.99	-0.09

[a] ACS Catal. 2018, 8, 9, 7952–7960.
[b] J. Am. Chem. Soc. 2025, 147, 2, 1624–1630.

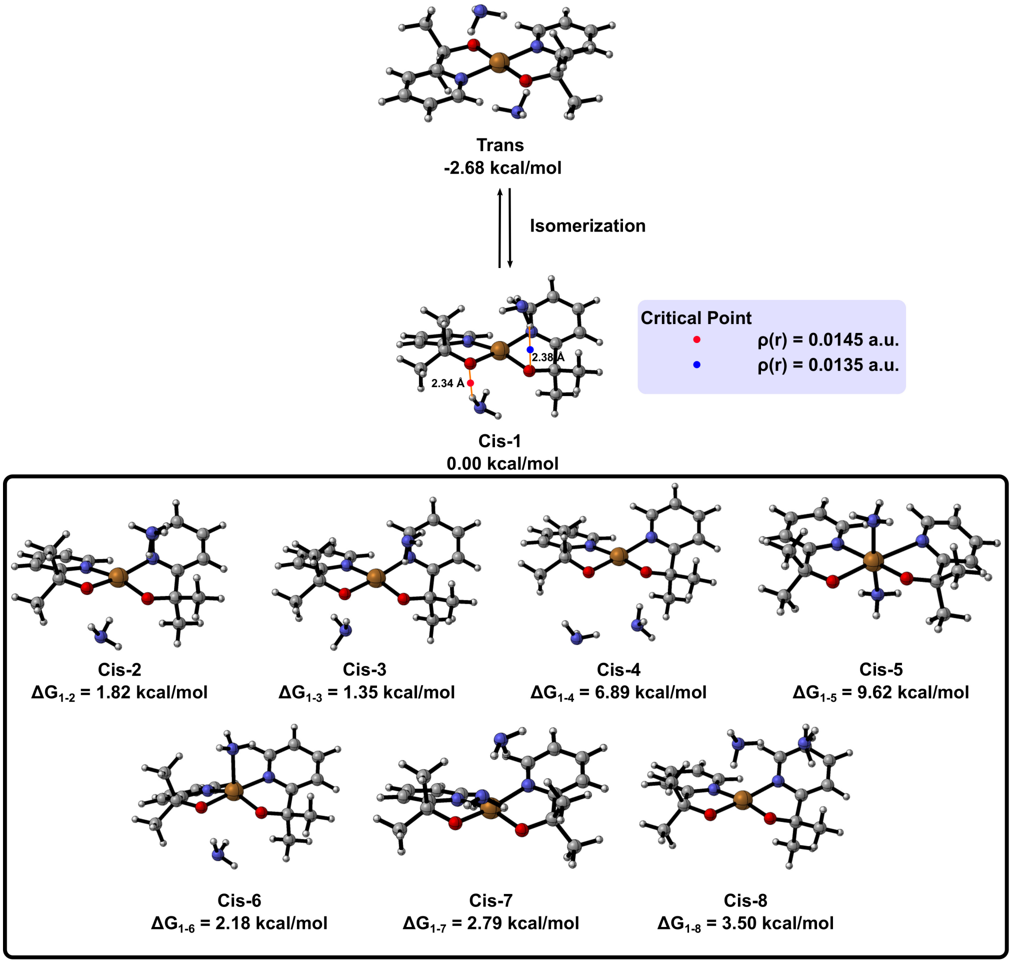

Fig. S1: Different ammonia coordination and organization modes in $Cu(pyalk)_2$ structures

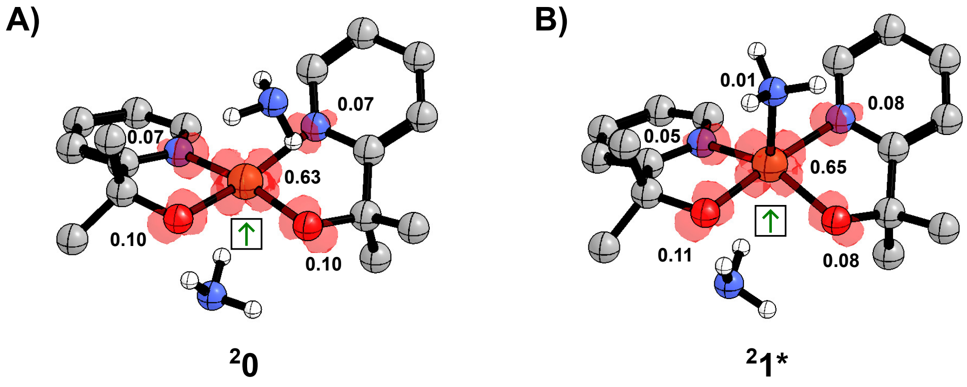

Fig. S2: Spin density isosurface of species ^{2}**0** and ^{2}**1***.

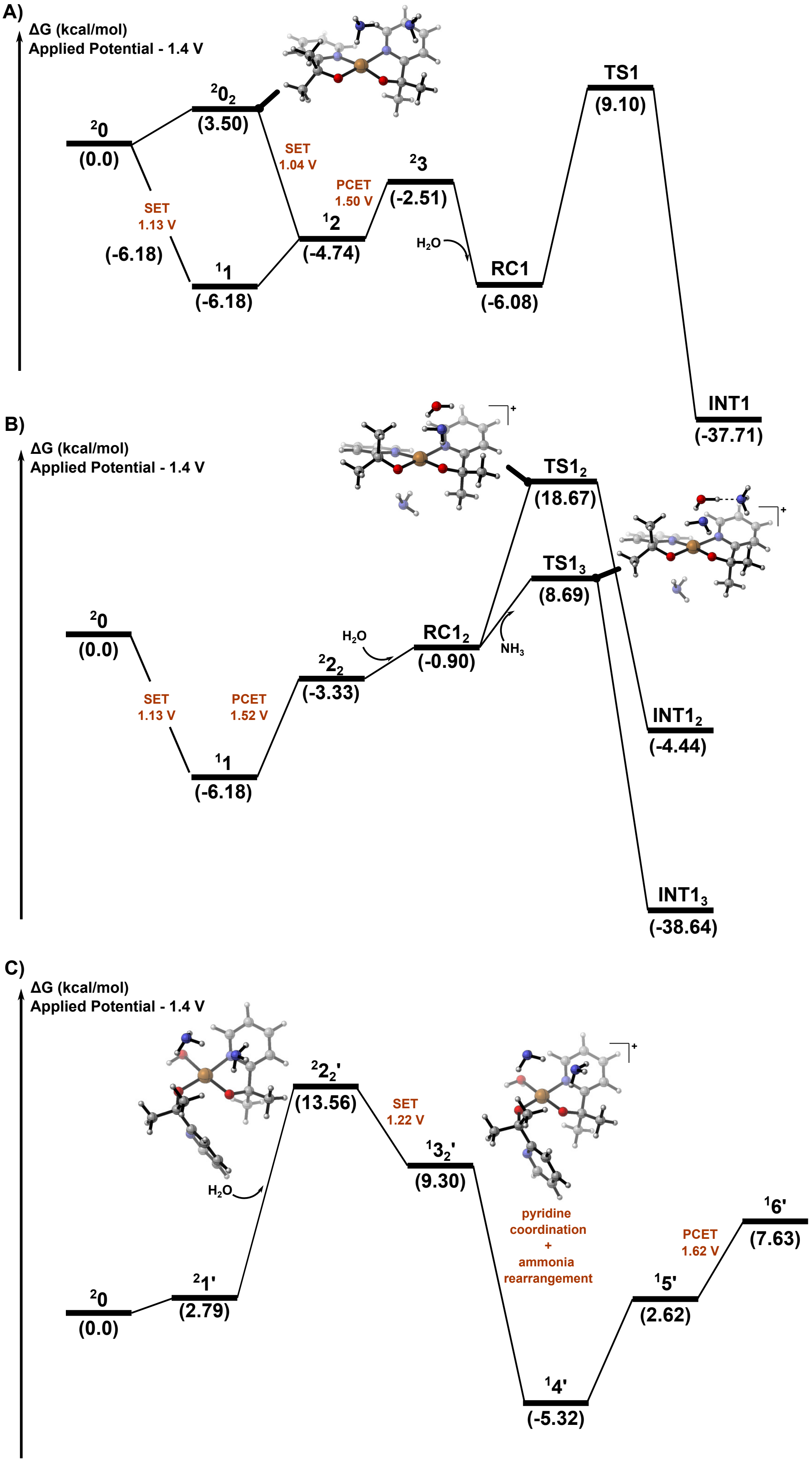

Fig. S3: Gibbs free energy profiles for alternative pathways: A) Ammonia reorganization prior to metal SET, B) transition states along the WNA Pathway, and C) initial water coordination in the ANA mechanism.

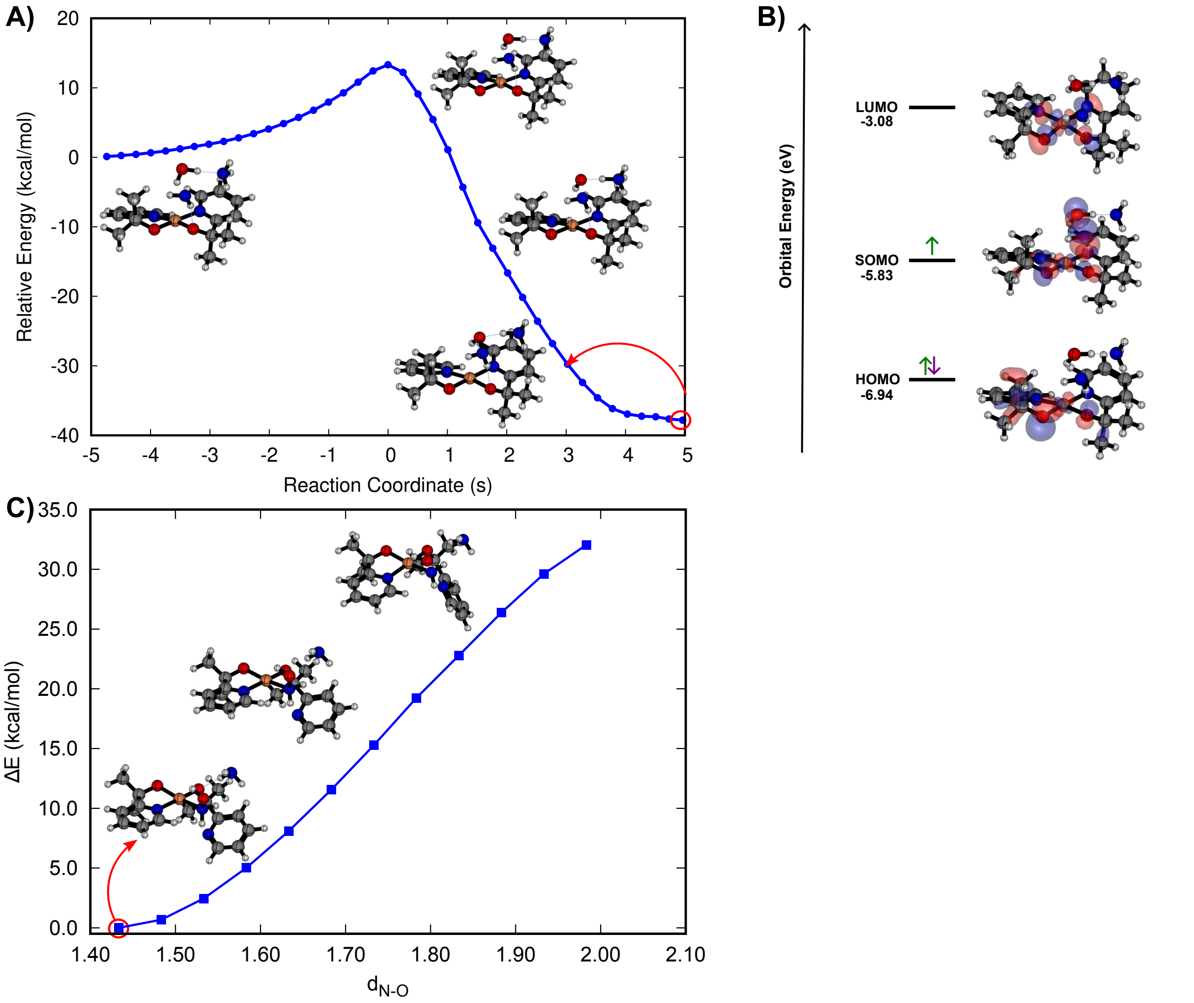

Fig. S4: A) Intrinsic reaction coordinate (IRC) profile for **TS1** connecting to **INT1**. B) Three-dimensional representation of molecular orbital of **TS1** transition state. C) A relaxed scan initiated from ^{2}**5*** for the N–O bond dissociation showing a barrierless process.

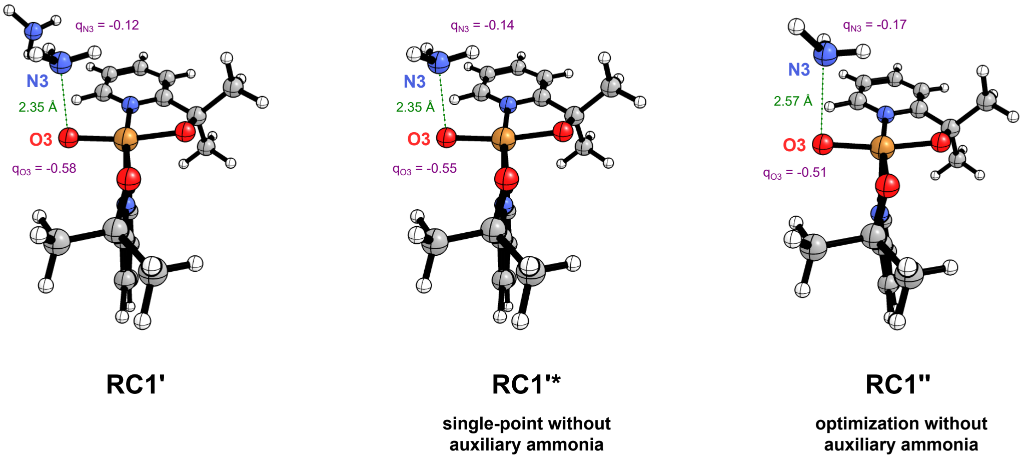

Fig. S5: Mulliken charge analysis for **RC2′** emphasizing the role of auxiliary ammonia.

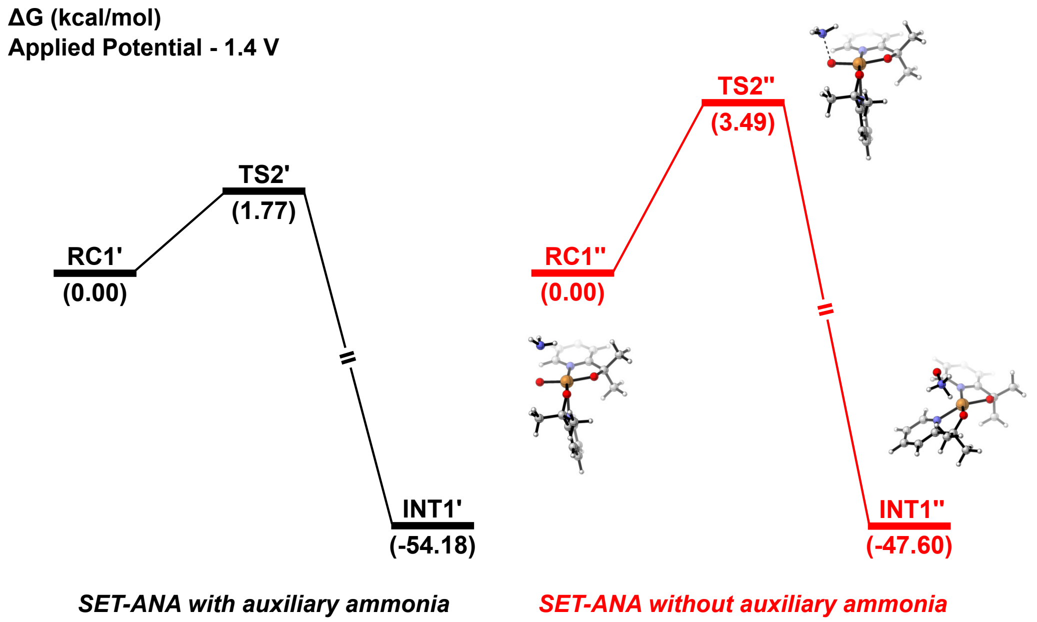

Fig. S6: SET-ANA energy profiles with (in black) and without (in red) auxiliary ammonia.

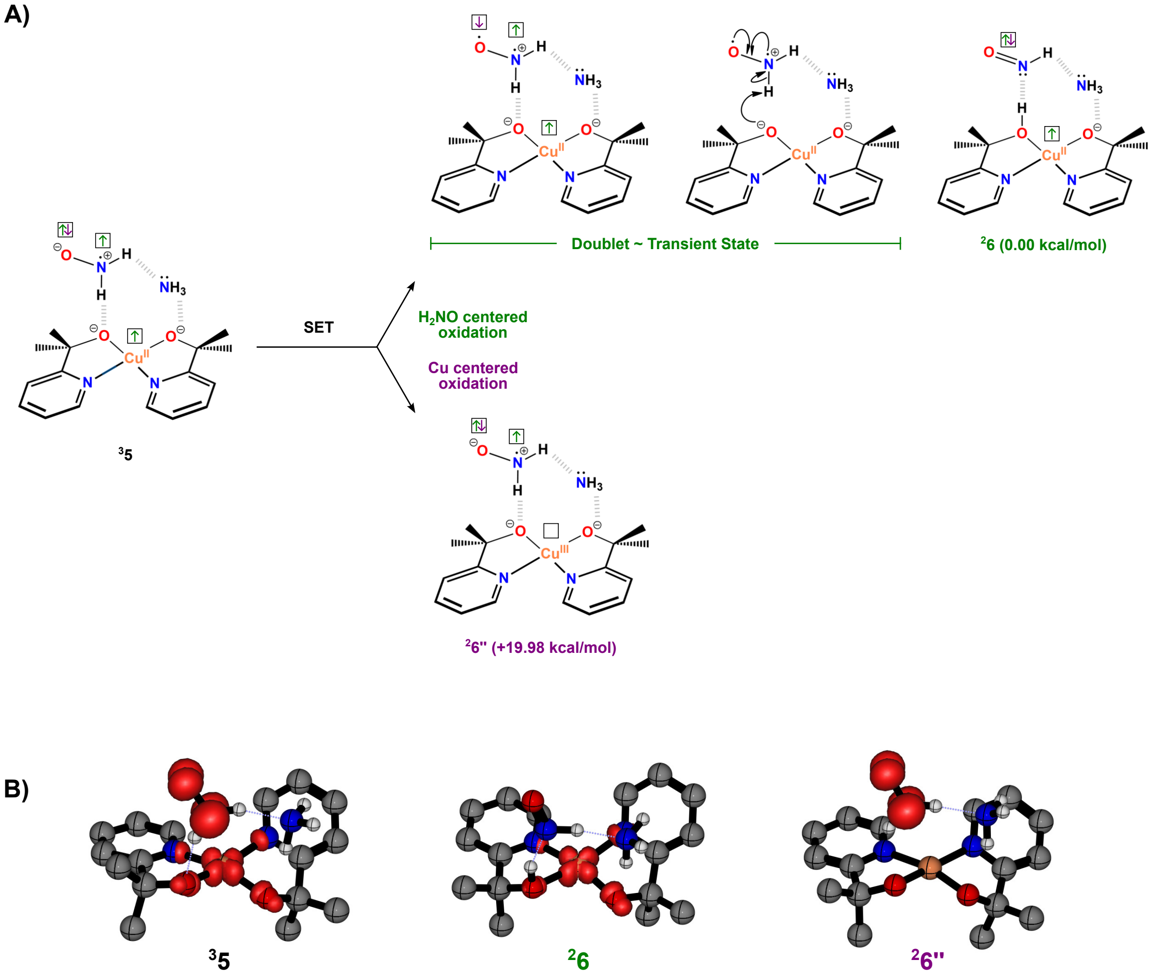

Fig. S7: A) Two-dimensional representations and of the proposed transient species involved the transformation from species $^{3}\mathbf{5}$ to $^{2}\mathbf{6}/^{2}\mathbf{6}''$ and B) the corresponding spin densities distributions.

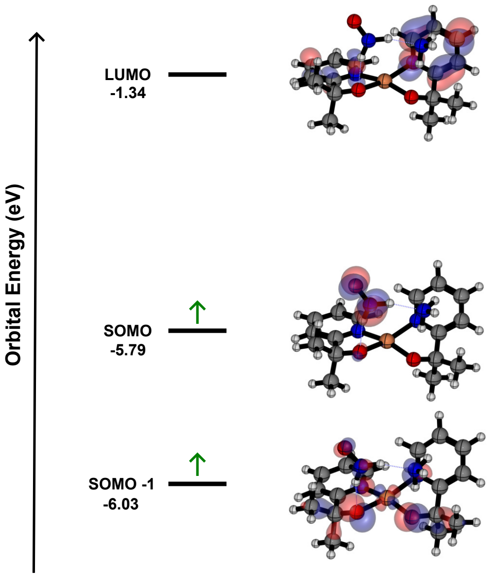

Fig. S8: Three-dimensional representation of molecular orbital of $^{3}\mathbf{5}$ specie.

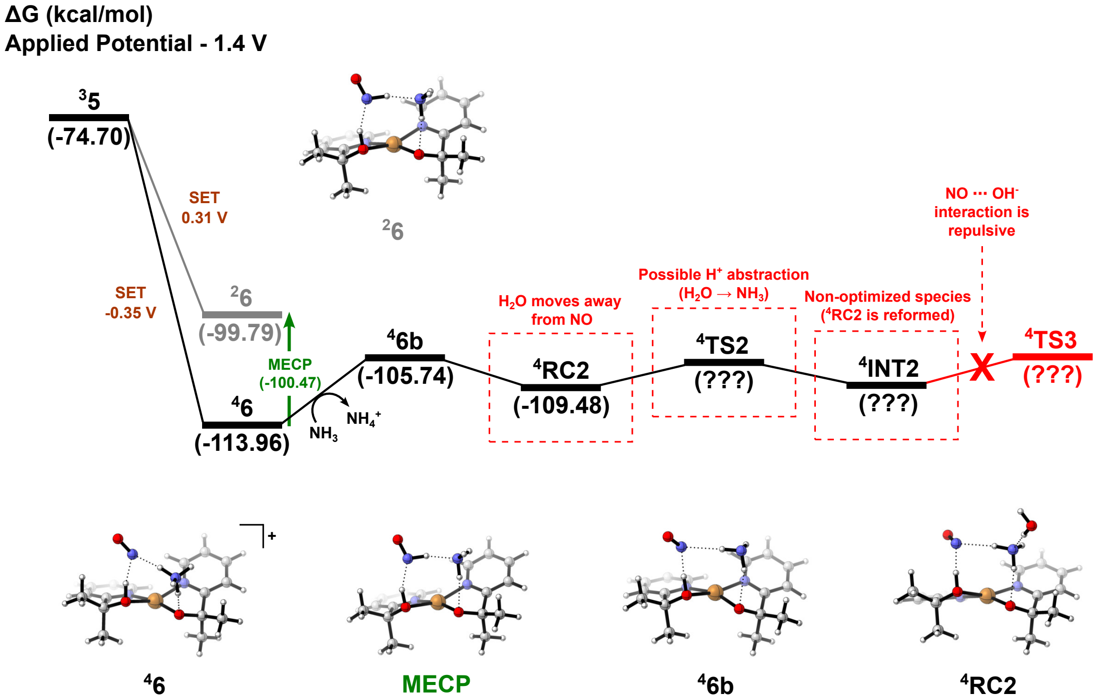

Fig. S9: Possible quartet PES for the second N−O coupling.

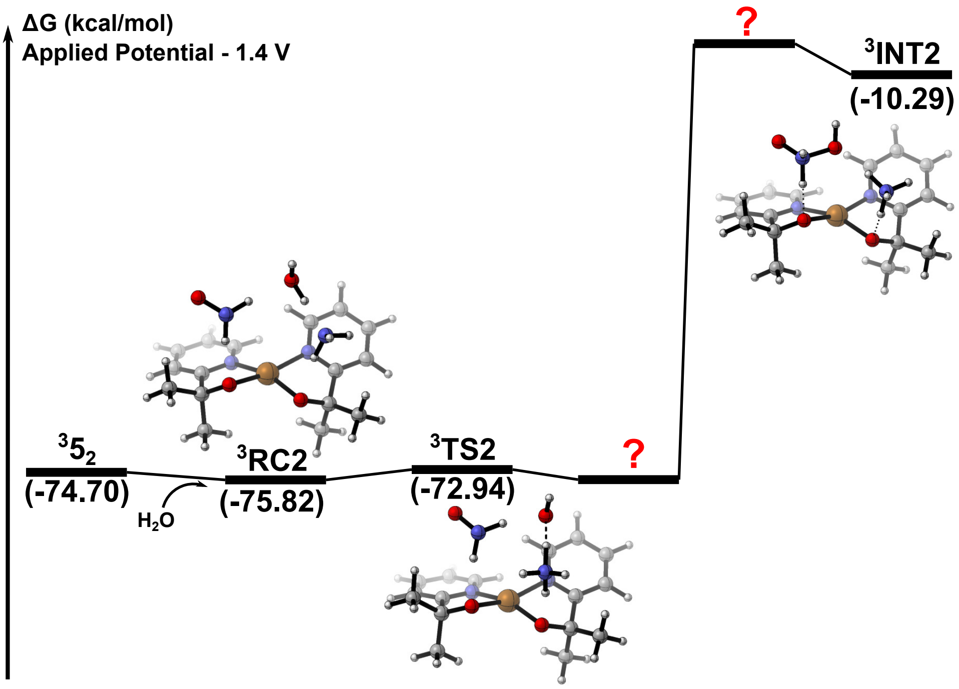

Fig. S10: Gibbs free energy profiles for alternative pathways of the initial step leading to formation of the second N–O bond on the triplet surface.

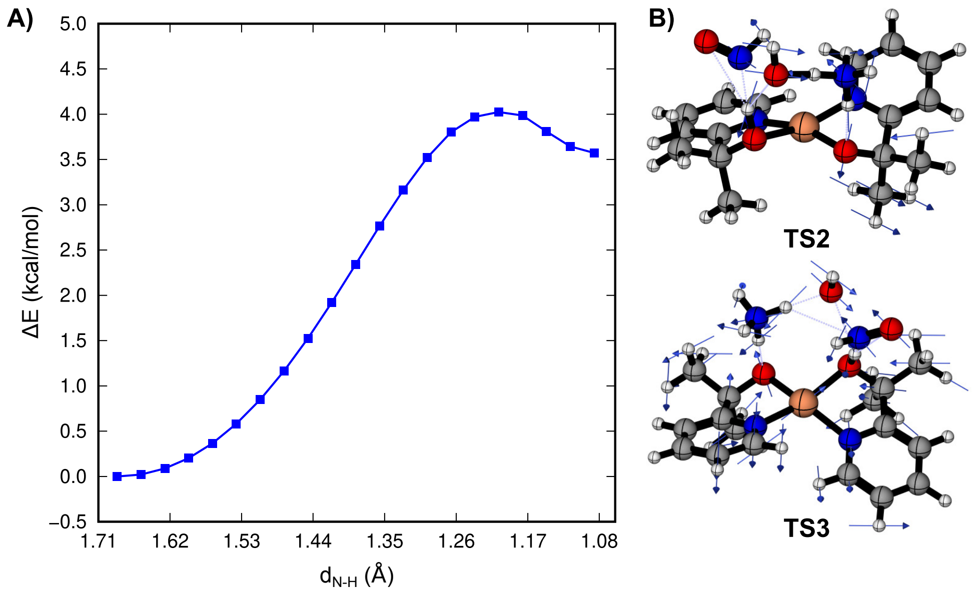

Fig. S11: A) A relaxed scan initiated from TS2 connecting RC2 and INT2 shows a lower electronic energy barrier; B) displacement vectors for the imaginary vibrational mode of **TS2** and **TS3**.

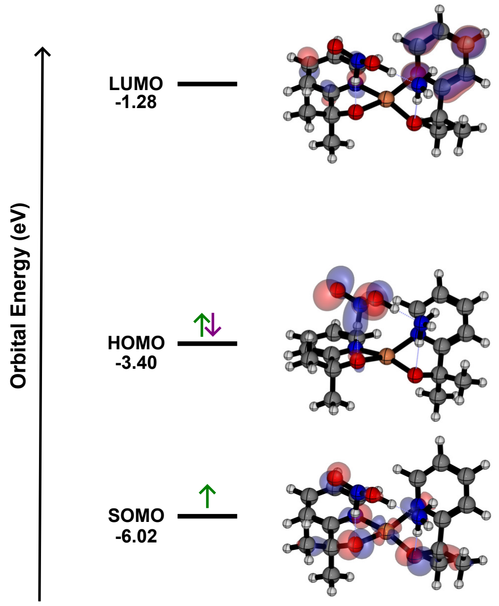

Fig. S12: Three-dimensional representation of molecular orbital of **^{2}7** specie.

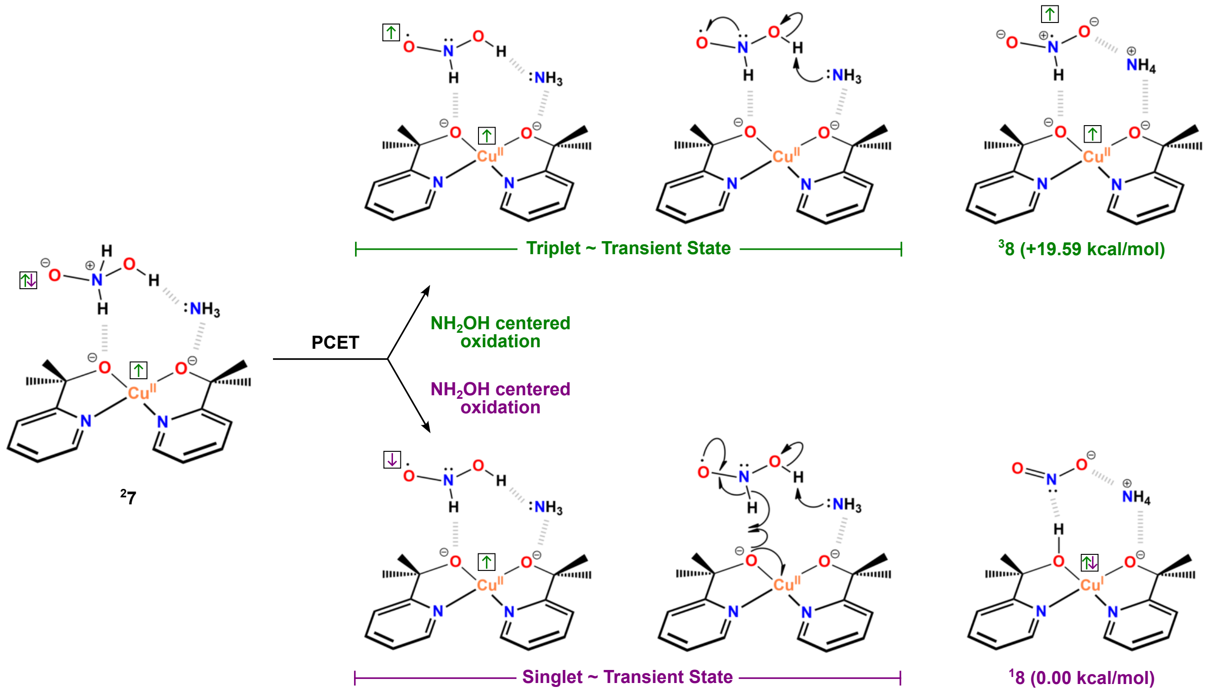

Fig. S13: Two-dimensional representations of the proposed transient species involved the transformation from species **^{2}7** to **^{3}8**/**^{1}8**.

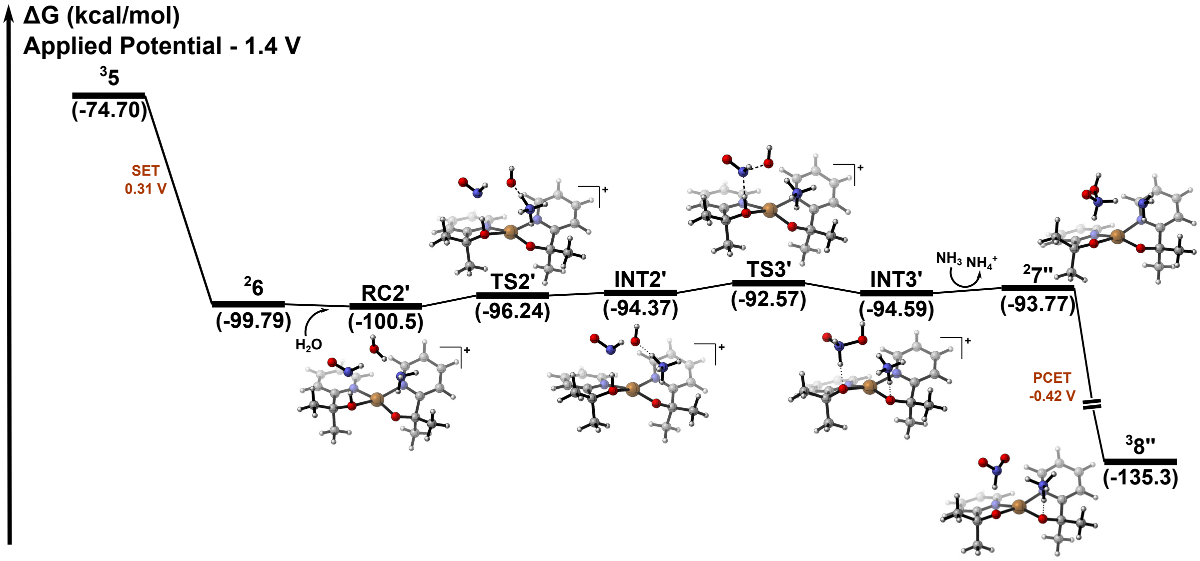

Fig. S14: Gibbs free energy profiles for alternative pathways for the formation of the second N–O bond, in which the water molecule approaches the metal complex from above rather than from the front.

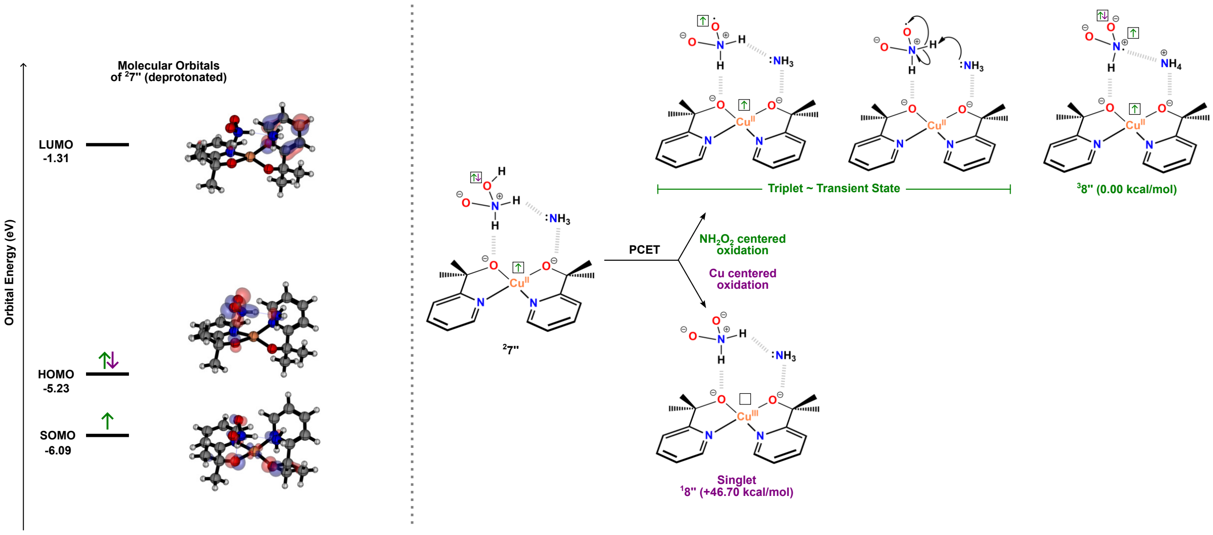

Fig. S15: Molecular orbitals of $^{2}7''$, and two-dimensional representations of this proposed transient species involved in the transformation from $^{2}7''$ to $^{3}8''/^{1}8''$.

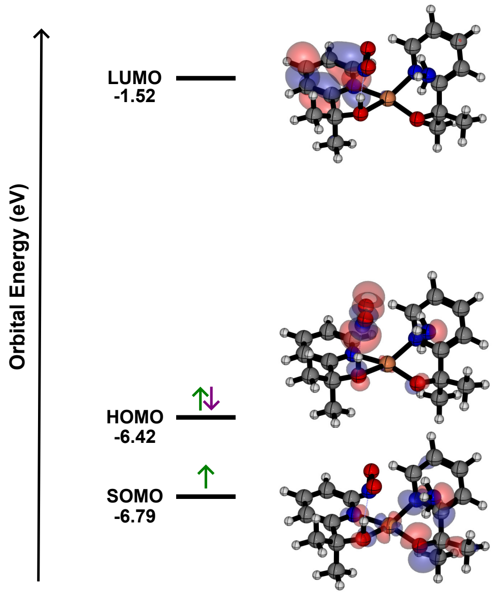

Fig. S16: Three-dimensional representation of molecular orbital of species ^{2}**9**.

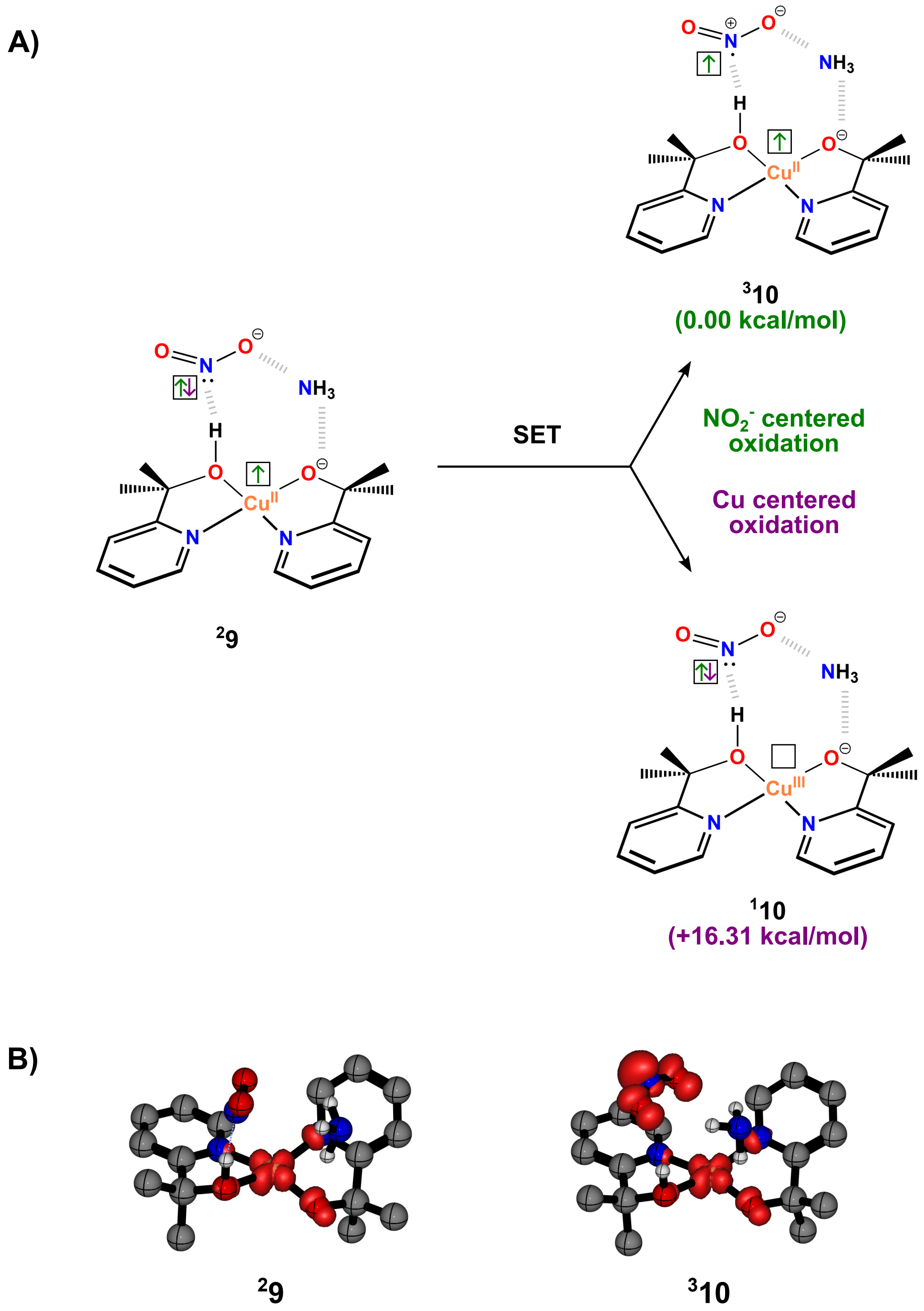

Fig. S17: Two-dimensional representations of the proposed transient species involved the transformation from species **^{2}9** to **^{3}10**/**^{1}10**.

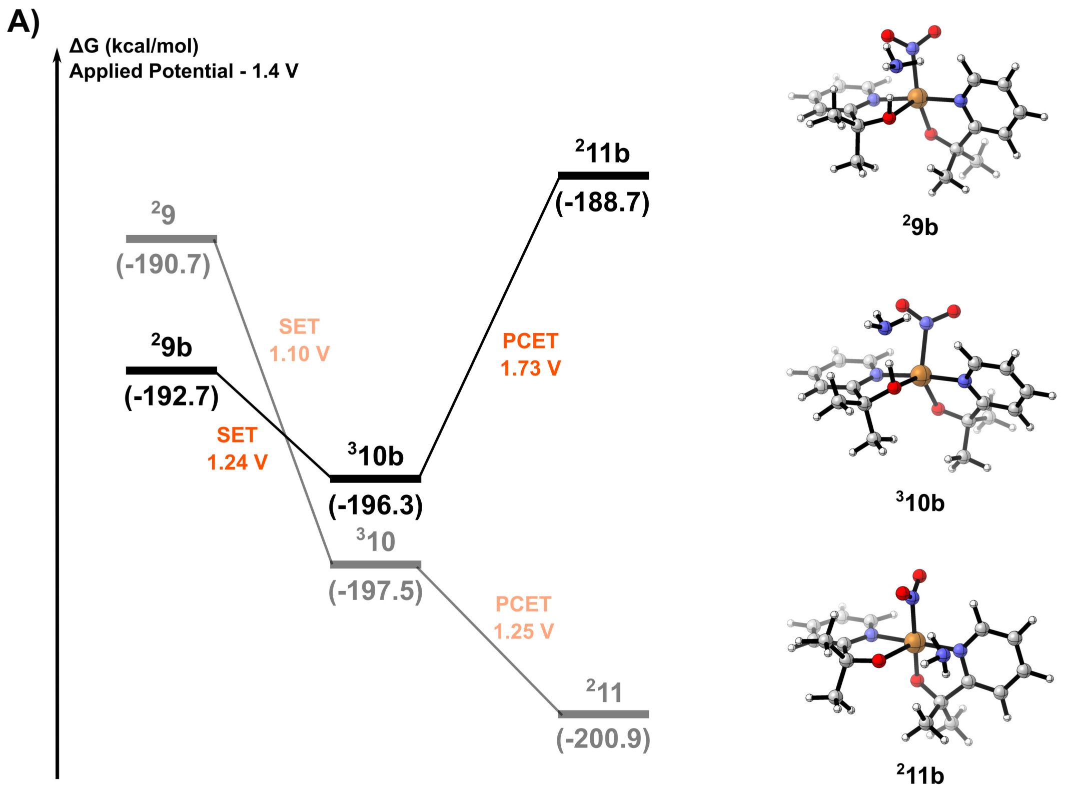

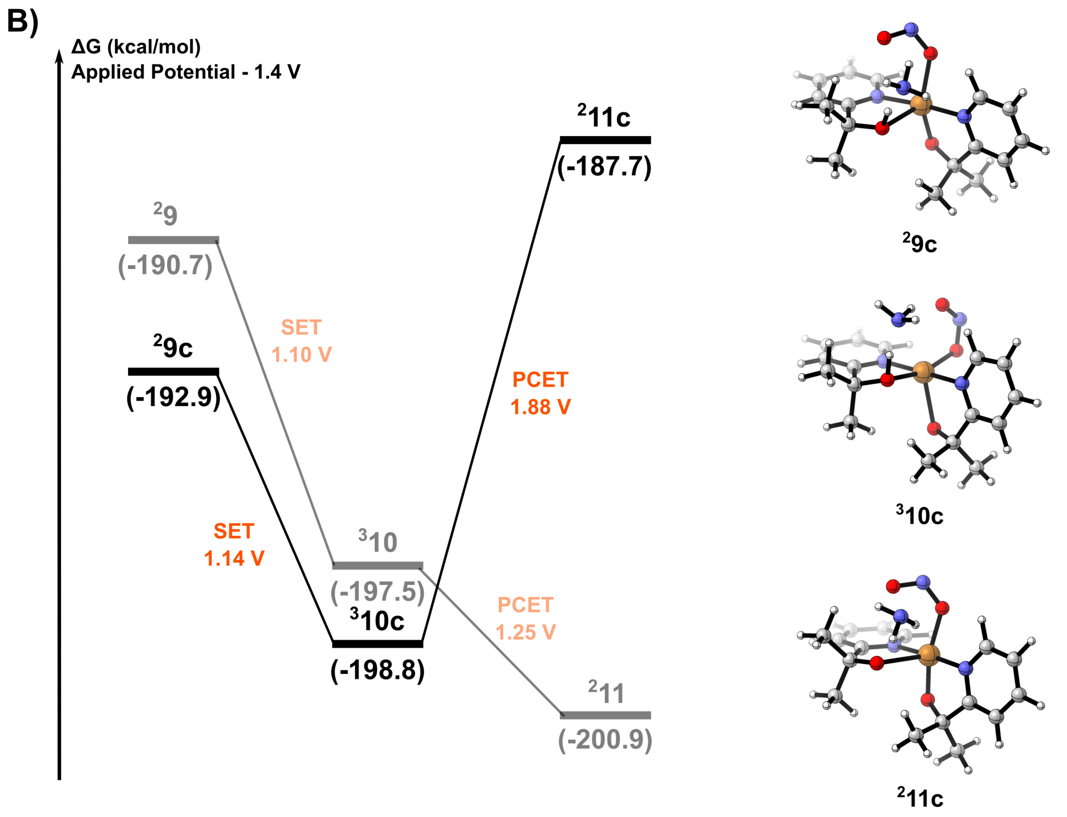

Fig. S18: Gibbs free energy profiles for alternative pathways of the initial step that leads to NO_2^- release, starting from the equatorial isomer: A) N-bound and B) O-bound coordination to the metal center.
